# Exchanging Replicas with Unequal Cost, Infinitely and Permanently

**DOI:** 10.1021/acs.jpca.2c06004

**Published:** 2022-11-17

**Authors:** Sander Roet, Daniel T. Zhang, Titus S. van Erp

**Affiliations:** Department of Chemistry, Norwegian University of Science and Technology (NTNU), N-7491Trondheim, Norway

## Abstract

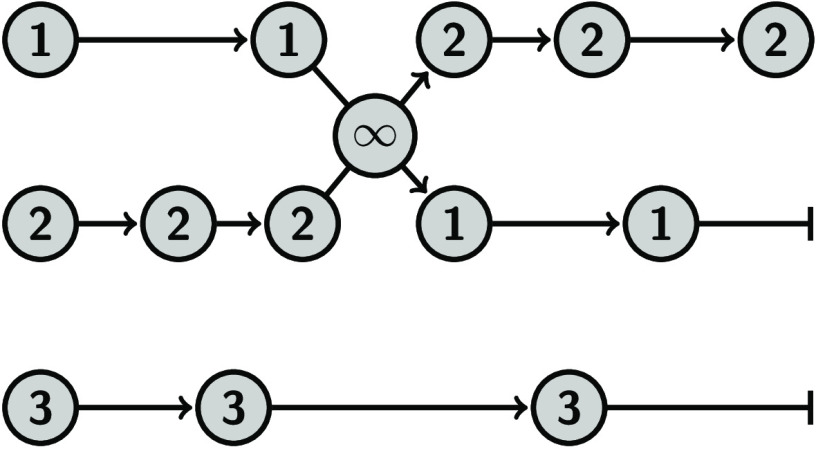

We developed a replica exchange method that is effectively parallelizable even if the computational cost of the Monte Carlo moves in the parallel replicas are considerably different, for instance, because the replicas run on different types of processor units or because of the algorithmic complexity. To prove detailed-balance, we make a paradigm shift from the common conceptual viewpoint in which the set of parallel replicas represents a high-dimensional superstate, to an ensemble-based criterion in which the other ensembles represent an environment that might or might not participate in the Monte Carlo move. In addition, based on a recent algorithm for computing permanents, we effectively increase the exchange rate to infinite without the steep factorial scaling as a function of the number of replicas. We illustrate the effectiveness of this replica exchange methodology by combining it with a quantitative path sampling method, replica exchange transition interface sampling (RETIS), in which the costs for a Monte Carlo move can vary enormously as paths in a RETIS algorithm do not have the same length and the average path lengths tend to vary considerably for the different path ensembles that run in parallel. This combination, coined ∞RETIS, was tested on three model systems.

## 1 Introduction

The Markov chain Monte Carlo (MC) method is one of the most important numerical techniques for computing averages in high-dimensional spaces, like the configuration space of a many-particle system. The approach has applications in a wide variety of fields ranging from computational physics, theoretical chemistry, economics, and genetics. The MC algorithm effectively generates a selective random walk through state space in which the artificial steps are designed to ensure that the frequency of visiting any particular state is proportional to the equilibrium probability of that state. The Metropolis^1^ or the more general Metropolis–Hastings^2^ algorithms are the most common approaches for designing such random steps (MC moves) based on the detailed-balance principle. That is, the MC moves should be constructed such that the number of transitions from an old state *s*^*o*^ to a new state *s*^*n*^ is exactly balanced by the number of transitions from the new to the old state: ρ(*s*^(*o*)^)π(*s*^(*o*)^ → *s*^(*n*)^) = ρ(*s*^(*n*)^) π(*s*^(*n*)^ → *s*^(*o*)^), where ρ(·) is the state space equilibrium probability density and π(·) are the probabilities to make a transition between the two states given the set of possible MC moves. Further, the transition is split into a generation and an acceptance/rejection step such that π(*s* → *s*′) = *P*_gen_(*s* → *s*′) *P*_acc_(*s* → *s*′). In the case that the sampled state space is the configuration space of a molecular system at constant temperature, *P*_gen_ might relate to moving a randomly picked particle in a random direction over a small random distance, and ρ(*s*) is proportional to the Boltzmann weight e^–β*E*(*s*)^, with β = 1/*k*_B_*T* the inverse temperature, *k*_B_ the Boltzmann constant, and *E*(*s*) the state’s energy.

The Metropolis–Hastings algorithm takes a specific solution for the acceptance probability
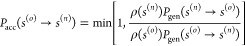
1

The generation probabilities will cancel in the above expression if they are symmetric, *P*_gen_(*s* → *s*′) = *P*_gen_(*s*′ → *s*) as in the less generic Metropolis scheme. At each MC step, the new state is either accepted or rejected based on the probability above. In case of a rejection, the old state is maintained and resampled. This scheme obeys detailed-balance. In addition, if the set of MC moves are ergodic, equilibrium sampling is guaranteed. When ergodic sampling, even if mathematically obeyed, is slowed down by a rough (free) energy landscape, replica exchange MC becomes useful.

Replica exchange MC (or replica exchange molecular dynamics) is based on the idea to simulate several copies of the system with different ensemble definitions,^3−5^ most commonly ensembles with increasing temperature (parallel tempering). By performing “swaps” between adjacent replicas, the low-temperature replicas gain access to the broader space region that are explored by the high-temperature replicas. The detailed-balance and corresponding acceptance–rejection step can be derived by viewing the set of states in the different ensembles (replicas) as a single high-dimensional superstate *S* = (*s*_1_, *s*_2_, ···, *s*_*N*_) representing the system in a set of *N* independent “parallel universes”. The Metropolis scheme applied to the superstate yields
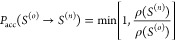
2in which the probability of the superstate equals
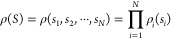
3where ρ_*i*_(·) is the specific probability density of ensemble *i*. For example, the move that attempts to swap the first two states, implying *S*^*o*^ = (*s*_1_, *s*_2_, ···, *s*_*N*_) and *S*^*n*^ = (*s*_2_, *s*_1_, ···, *s*_*N*_), will be accepted with a probability
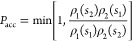
4

In a replica exchange simulation, swapping moves and standard MC or MD steps are applied alternately. Parallel computing will typically distribute the same number of processing units per ensemble to carry out the computationally intensive standard moves. The swapping move is cheap but requires that the ensembles involved in the swap have completed their previous move. If the standard moves in each ensemble require different computing times, then several processing units have to wait for the slow ones to finish.

If the disbalance per move is relatively constant, then the replicas could effectively be made to progress in cohort by trying to differentiate the number of processing units per ensemble or the relative frequency of doing replica exchange versus standard moves per ensemble. However, this disbalance is not constant in several MC methods, such as with configurational bias MC^6−8^ or path sampling.^9^ The number of elementary steps to grow a polymer in configurational bias MC obviously depends on the polymer’s length that is being grown, but also early rejections lead to a broad distribution of the time it takes to complete a single MC move even in uniform polymer systems. Analogously, the time required to complete an MC move in path sampling simulations will depend on the length of the path being created. Other examples of complex Monte Carlo methods with a fluctuating CPU cost per move are cluster Monte Carlo algorithms^10^ and event-chain Monte Carlo.^11,12^

We will show that the standard acceptance eqs 1 and 4 can be applied in a parallel scheme in which ensembles are updated irregularly in time and the average frequency of MC moves is different for the ensembles. In addition, we show that we can apply an infinite swapping^13^ scheme between the available ensembles. For this, we develop a new protocol based on the evaluation of permanents that circumvents the steep factorial scaling. This last development is also useful for standard replica exchange.

## 2 Methods

### 2.1 Finite Swapping

In the following, we will assume that we have two types of MC moves. One move that is CPU-intensive and can be carried out within a single ensemble, and replica exchange moves between ensembles which are relatively cheap to execute. The CPU-intensive move will be carried out by a single worker (one processor unit, one node, or a group of nodes) and these workers perform their task in parallel on the different ensembles. One essential part of our algorithm is that we have less workers than ensembles such that whenever the worker is finished and produced a new state for one ensemble, this state can directly be swapped with the states of any of the available ensembles (the ones not occupied by a worker). After that, the worker will randomly switch to another unoccupied ensemble for performing a CPU-intensive move.

In its most basic form, the algorithm consists of the following steps:

1. Define *N* ensembles and let ρ_*i*_(·) be the probability distribution of ensemble *i*. We also define *P*_RE_ which is the probability of doing a replica exchange move.
2. Assign *K* < *N* workers (processing units) to *K* of the *N* ensembles for performing a CPU-intensive MC move. Each ensemble is at all times occupied by either 1 or 0 workers. The following steps are identical for all of the workers.
3. If the worker is finished with its MC move in ensemble *i*, the new state is accepted or rejected according to eq 1 (with ρ_*i*_ for ρ). Ensemble *i* is updated with the new state (or by resampling the old state in case of rejection) and is then considered to be free.
4. Take a uniform random number ν between 0 and 1. If ν > *P*_RE_, go to step 7.
5. Among the free ensembles, pick a random pair (*i*, *j*).
6. Try to swap the states of ensembles *i* and *j* using eq 4 (with labels *i*, *j* instead of 1, 2). Update ensembles *i*, *j* with the swapped state or the old state in case of a rejection. Return to step 4.
7. Select one of the free ensembles at random and assign the worker to that ensemble for performing a new standard move. Go to step 3.

In this algorithm, ensembles are not updated in cohort like in standard replica exchange, but updates occur at irregular intervals. In addition, the different ensemble conditions can result in systematic differences in the number of states that are being created over time. To prove that the above scheme actually samples the correct distributions requires a fundamentally new conceptual view as the superstate picture is no longer applicable. Despite that the algorithm uses the same type of eqs 1 and 4, as one would use in standard replica exchange, it does not rely on eqs 2 and 3 that are no longer valid. In the SI, we provide a proof from the individual ensemble’s perspective in which the other ensembles provide an “environment”  that might, or might not, participate in the move of the ensemble considered. By doing so, we no longer require that the number of transitions from old to new, *S*^(*o*)^ → *S*^(*n*)^, is the same as from new to old, *S*^(*n*)^ → *S*^(*o*)^. Instead, by writing , from ensemble 1’s perspective, we have that the number of  transitions should be equal to the number of  transitions when the standard move is applied where  refers to *any* new environment. In the SI, we show a similar detailed-balance condition for the replica exchange moves. At step 6 we sample only ensemble *i* and *j* or, alternatively, all free ensembles get a sample update. This would mean resampling the existing state of those not involved in a swap (“null move”). This makes the approach more similar to the superstate sampling albeit using only free ensembles, as described in the SI. The null move does not reduce the statistical uncertainty, but we mention it here as it makes it easier to explain the infinite swapping approach. But for the detailed-balance conditions to be valid it is imperative that occupied ensembles are not sampled.

An essential aspect of the efficiency of our algorithm is that the number of workers *K* is less than the number of ensembles *N*. The case *K* = *N* is valid but would reduce the number of replica exchange moves to zero as only one ensemble is free at the maximum. Reducing the *K*/*N* ratio will generally imply a higher acceptance in the replica exchange moves as we can expect a higher number of free ensembles whose distributions have significant overlap. What gives the optimum number of workers is therefore a nontrivial question that we will further explore in Section 4. However, for case *K* < N we can maximize the effect of the replica exchange moves by taking the *P*_RE_ parameter as high as possible. In fact, we can simulate the effect of the limit *P*_RE_ → 1 without having to do an infinite number of replica exchange moves explicitly. This leads to an infinite swapping^13^ version of our algorithm.

### 2.2 Infinite Swapping

If in the previously described algorithm we take *P*_RE_ = 1 – δ, we will loop through steps 4–6 for many iterations (*n*_it_ = ∑_*n* = 0_^∞^*n*(1 – δ)^*n*^δ = 1/δ in the limit δ → 0) before getting to step 7. When δ vanishes and *n*_it_ becomes infinitely large, we expect that all possible swaps will be executed an infinite number of times. Since the swaps obey detailed-balance between unoccupied ensembles, these will essentially sample the distribution of eq 3 (for the subset S* of unoccupied ensembles). Hence, when the loop is exited, each possible permutation σ ∈ S* has been sampled *n*_it_ × ρ(σ)/∑_σ_ρ(σ) times. By lumping all the times that the same permutation was sampled and normalizing by division with *n*_it_, we simply sample all of the possible permutations in one go using fractional weights that sum up to 1. This is then the only sampling step, as the single update in step 3 can be skipped due to its negligible 1/*n*_it_ weight.

The idea of doing an “infinite number” of swapping moves has been proposed before,^13−16^ but here we give a different flavor to this approach by a convenient reformulation of the problem into permanents that allows us to beat the steep factorial scaling reported in earlier works.^13^ The permanent formulation goes as follows. Suppose that after step 3, there are four free ensembles (we name them *e*_1_, *e*_2_, *e*_3_, *e*_4_) containing four states (*s*_1_, *s*_2_, *s*_3_, *s*_4_). Which state is in which ensemble after this step is irrelevant. We can now define a weight-matrix *W*
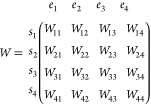
where *W*_*ij*_ ∝ ρ_*j*_(*s*_*i*_). Essential to our approach is the computation of the permanent of the *W*-matrix, perm(*W*), and that of the *W*{*ij*}-matrices in which the row *i* and column *j* are removed.

The permanent of a matrix is similar to the determinant but without alternating signs. We can, henceforth, write perm(*W*) = ∑_*j*=1_^4^*W*_1*j*_perm(*W*{1*j*}). As the permanent of the 1 × 1 matrix is obviously equal to the single matrix value, the permanent of arbitrary dimension could in principle be solved recursively using this relation. Based on the permanents of *W*, we will construct a probability matrix *P*
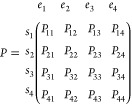
where *P*_*ij*_ is the chance to find state *s*_*i*_ in ensemble *e*_*j*_. As for each permutation each state is in one ensemble and each ensemble contains one state, the *P*-matrix is bistochastic: both the columns and the rows sum up to 1. If we consider *S*_ij_^*^ the set of permutations in which state *s*_*i*_ is in *e*_*j*_, we can write *P*_*ij*_ = ∑_σ∈*S*_ij_^*^_ ρ(σ)/∑_σ′∈S_*ρ(σ′). We can, however, also use the permanent representation in which
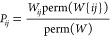
5So far we have not won anything as computing the permanent via the recursive relation mentioned above has still the factorial scaling. The Gaussian elimination approach, which allows an order  computation for determinants of *n* × *n* matrices, will not work for permanents as only some but not all row and column operations have the same effect to a permanent as to a determinant. One can for instance swap rows and columns without changing the permanent. Multiplying a row by a nonzero scalar multiplies the permanent by the same scalar. Hence, this will not affect the P-matrix based on eq 5. Unlike the determinant, adding or subtracting to a row a scalar multiple of another row, an essential part of the Gaussian elimination method, does change the permanent. This makes the permanent computation of a large matrix excessively more expensive than the computation of a determinant. Yet, recent algorithms based on the Balasubramanian-Bax-Franklin-Glynn (BBFG) formula^17−20^ scale as . This means that the computation of the full *P*-matrix scales as , which seems still steep but is nevertheless a dramatic improvement compared to factorial scaling.

For our target time of 1 second, for instance, we could only run the algorithm up to *N* = 7 in the factorial approach, while we reach *N* = 12 in the BBFG method using a mid-to-high-end laptop (DELL XPS 15 with an Intel Core i7-8750H). If matrix size of *N* = 20 is the target, the BBFG method can perform a full *P*-matrix determination in ∼711 s, while it would take ∼15.3 × 10^6^ years in the factorial approach. The BBFG method is the fastest completely general solution for the problem of computing a *P*-matrix from any *W*-matrix. For several algorithms, the *W*-matrix has special characteristics that can be exploited to further increase efficiency. For instance, if by shuffling the rows and columns the *W*-matrix can be made into a block form, where squared blocks at the diagonal have only zeros at their right and upper side, the permanent is equal to the product of the block’s permanents. For instance, if *W*_14_ = *W*_24_ = *W*_34_ = 0, we have two blocks, 3 × 3 and 1 × 1. If *W*_13_ = *W*_14_ = *W*_23_ = *W*_24_ = 0, we can identify two blocks of 2 × 2, etc. Identification of blocks can hugely decrease the computation of a large permanent. Another speed-up can be made if all rows in the *W*-matrix are a sequence of ones followed by all zeros, or can be made into that form after the previously mentioned column and row operations. This makes an order  approach possible. We will further discuss this in Section 3.1.

The infinite swapping approach changes the aforementioned algorithm from step 3:

3 If the worker is finished with its MC move in a specific ensemble, the new state is accepted or rejected (but not yet sampled) according to eq 1. The ensemble is free.
4 Determine the *W*-matrix based on all unoccupied ensembles, calculate the *P*-matrix based on eq 5, and update all of the unoccupied ensembles by sampling all free states with the fractional probabilities corresponding to the columns in the *P*-matrix.
5 Pick randomly one of the free ensembles *e*_*j*_.
6 Pick one of the available states (*s*_1_, *s*_2_, ···) based on a weighted random selection in which state *s*_*i*_ has a probability of *P*_*ij*_ to be selected.
7 The worker is assigned to do a new standard move in ensemble *e*_*j*_ based on previous state *s*_*i*_. Go to step 3.

## 3 Application: ∞RETIS

Replica Exchange Transition Interface Sampling (RETIS)^21,22^ is a quantitative path sampling algorithm in which the sampled states are short molecular trajectories (paths) with certain start and end conditions, and a minimal progress condition. New paths are being generated by a Monte Carlo move in path space, such as the shooting move^23^ in which a randomly selected phase point of the previous path is randomly modified and then integrated backward and forward in time by means of molecular dynamics (MD). The required minimal progress increases with the rank of the ensemble such that the final ensemble contains a reasonable fraction of transition trajectories. The start and end conditions, as well as the minimal progress, are administered by the crossings of interfaces (λ_0_, λ_1_, ···, λ_*M*_) with λ_*k*+1_ > λ_*k*_, that can be viewed as nonintersecting hypersurfaces in phase space having a fixed value of the reaction coordinate. A MC move that generates a trial path not fulfilling the path ensemble’s criteria is always rejected. RETIS defines different path ensembles based on the direction of the paths and the interface that has to be crossed, but all paths start by crossing λ_0_ (near the reactant state/state *A*) and they end by either crossing λ_0_ again or reaching the last interface λ_*M*_ (near the product state/state *B*). There is one special path ensemble, called [0^–^], that explores the left side of λ_0_, the reactant well, while all other path ensembles, called [*k*^+^] with *k* = 0, 1, ··· *M* – 1, start by moving to the right from λ_0_ reaching at least λ_*k*_.

A central concept in RETIS is the so-called overall crossing probability, the chance that a path that crosses λ_0_ in the positive direction reaches λ_*M*_ without recrossing λ_0_. It provides the rate of the process when multiplied with the flux through λ_0_ (obtained from the path lengths in [0^–^] and [0^+^]^22^) and is usually an extremely small number. The chance that any of the sampled paths in the [0^+^] path ensemble crosses λ_*M*_ is generally negligible, but a decent fraction of those (∼0.1–0.5) will cross λ_1_ and some even λ_2_. Likewise, paths in the [*k*^+^], *k* > 0, path ensembles have a much higher chance to cross λ_*k*+1_ than a [0^+^]-path as they already cross λ_*k*_. This leads to the calculation of *M* local conditional crossing probabilities, the chance to cross λ_*k*+1_ given λ_*k*_ was crossed for *k* = 0,1, ···, *M* – 1, whose product gives an exact expression for the overall crossing probability with an exponentially reduced CPU cost compared to MD.

The efficiency is further hugely improved by executing replica exchange moves between the path ensembles. These swaps are essentially cost-free since there is no need to simulate additional ensembles that are not already required. An accepted swapping move in RETIS provides new paths in two ensembles without the expense of having to do MD steps. The enhancement in efficiency is generally even larger than one would expect based on these arguments alone as path ensembles higher-up the barrier provide a similar effect as the high-temperature ensembles in parallel tempering. In addition, point exchange moves between the [0^–^] and [0^+^] ensembles are performed by exchanging the end and start points of these paths that are then continued by MD at the opposite site of the λ_0_ interface.

While TIS^24^ (without replica exchange) can run all path ensembles embarrassingly parallel, the RETIS algorithm increases the CPU-time efficiency but is difficult to parallelize and open source path sampling codes, like OpenPathSampling^25^ and PyRETIS,^26^ implement RETIS as a fully sequential algorithm. The path length distributions are generally broad with an increasing average path length as a function of the ensemble’s rank. This becomes increasingly problematic the more ensembles you have as they all have to wait for the slowest ensemble. This means that while RETIS will give the best statistics per CPU-hour, it might not give the best statistics in wall-time. Our parallel scheme can effectively deal with the unequal CPU cost of the replicas, which allows us to increase the wall-time efficiency with no or minimal reduction in CPU-time efficiency. On the contrary, our method does not give an equal distribution of the CPU-time over the different ensembles nor an equal number of samples per ensemble, leading to a smarter distribution of CPU hours. The CPU efficiency therefore even seems to improve slightly.

### 3.1 *W*-Matrix in RETIS

If there are *M* + 1 interfaces, λ_0_, λ_1_, ···, λ_*M*_, there are also *N* = *M* + 1 ensembles, [0^–^], [0^+^], [1^+^], ···, [(*M* – 1)^+^]. For *K* workers, the size of the *W*-matrix is, hence, either (*N* – *K* + 1) × (*N* – *K* + 1) or (*N* – *K*) × (*N* – *K*) as swappings are executed when 1 of the *K* workers is free, while the remaining *K* – 1 workers occupy path ensembles that are locked and do not participate in the swap. The smallest matrix occurs when one worker is occupying both [0^–^] and [0^+^] during the point exchange move, as described in the SI.

Paths can be represented by a sequence of time slices, the phase points visited by the MD trajectory. For a path of length *L* + 1, *X* = (*x*_0_, *x*_1_, ···, *x*_*L*_), the plain path probability density ρ(*X*) is given by the probability of the initial phase point times the dynamical transition probabilities to go from one phase point to the next: ρ(*X*) = ρ(*x*_0_)ϕ(*x*_0_ → *x*_1_)ϕ(*x*_1_ → *x*_2_) ··· ϕ(*x*_*L*–1_ → *x*_*L*_). Here, the transition probabilities depend on the type of dynamics (deterministic, Langevin, Nosé-Hoover dynamics, etc). The weight of a path within a specific path ensemble ρ_*j*_(*X*) can be expressed as the plain path density times the indicator function **1**_*e*_*j*__ and possibly an additional weight function *w*_*j*_(*X*): ρ_*j*_(*X*) = ρ(*X*) × **1**_*e*_*j*__(*X*) × *w*_*j*_(*X*). The indicator function equals 1 if path *X* belongs to ensemble *e*_*j*_. Otherwise, it is 0. The additional weight function *w*_*j*_(*X*) is part of the high-acceptance protocol that is used in combination with the more recent path generation MC moves such as stone skipping^27^ and wire fencing.^28^ Using these “high-acceptance weights”, nearly all of the CPU-intensive moves can be accepted as they are tuned to cancel the *P*_gen_-terms in the Metropolis–Hastings scheme, eq 1, and the effect of the nonphysical weights is undone in the analysis by weighting each sampled path with the inverse of *w*_*j*_(*X*).

While the path probability ρ(*s*_*i*_ = *X*) is difficult to compute, determining **1**_*j*_(*s*_*i*_) and *w*_*j*_(*s*_*i*_) is trivial. It is therefore a fortunate coincidence that we can replace *W*_*ij*_ = ρ_*j*_(*s*_*i*_) with
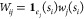
6because the *P*-matrix does not change if we divide or multiply a row by the same number, as mentioned in Section 2. Except for [0^–^], all path ensembles have the same start and end conditions and only differ with respect to the interface crossing condition. A path that crosses interface λ_*k*_ automatically crosses all lower interfaces λ_*l*<k_. Reversely, if the path does not cross λ_*k*_, it will not cross any of the higher interfaces λ_*l*>k_. This implies that if the columns of *W*_*ij*_ are ordered such that the first column (*e*_1_) is the first available ensemble from the sequence ([0^–^], [0^+^], [1^+^], ···, [(*M* – 1)^+^]), the second column (*e*_2_) is the second available ensemble, and so on, most rows will end with a series of zeros.

Reordering the rows with respect to the number of trailing zeros, almost always ensures that the *W*-matrix can be brought into a block form such that the permanent can be computed faster based on smaller matrices. In particular, if [0^–^] is part of the free ensembles, it will always form a 1 × 1 block as there is always one and no more than one available path that fits in this ensemble.

If high acceptance is not applied, we have *w*_*j*_(*X*) = 1 and each row in the *W*-matrix (after separating the [0^–^] ensemble if it is part of the free ensembles) is a sequence of ones followed by all zeros. The *W*-matrix can hence be represented by an array (*n*_1_, *n*_2_, *n*_3_, ··· *n*_*n*_), where each integer *n*_*i*_ indicates the number of ones in row *i*. As we show in the SI, the permanent of such a *W*-matrix is simply the product of (*n*_*i*_ + 1 – *i*): perm(*W*) = ∏_*i*_ (*n*_*i*_ + 1 – *i*). Further, the *P*-matrix can be constructed from the following order  method.

The first step is to order the rows of the *W*-matrix such that *n*_1_ ≤ *n*_2_ ≤ ··· ≤ *n*_*n*_. We then fill in the *P*-matrix from top to bottom for each row using
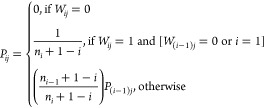
7The approach is extremely fast and allows the computation of *P*-matrices from a large *W*-matrix, up to several thousands, within a second of CPU-time. The above method applies whenever the rows of the *W*-matrix can be transformed into sequence of ones followed by all zeros. Besides RETIS without high acceptance, this would apply to other MC methods like subset sampling^29^ or umbrella sampling^30^ with semi-infinite rectangular windows.

## 4 Results and Discussion

To test our algorithms we ran three types of ∞RETIS simulations. First, a memoryless single variable stochastic (MSVS) process was simulated to mimic a RETIS simulation in which the average path length increases linearly with the rank of the ensemble. A “path” is created by drawing 2 random numbers where the first determines how much progress a path makes and the second determines the path length. These two outcomes are variable and depend on the rank of the ensemble such that the fictitious path in ensemble [*k*^+^] has a 0.1 probability to cross λ_*k*+1_ and has an average path length of approximately *k*/10 seconds (see Section 2). The worker is paused for a number of seconds equal to the path length before it can participate in replica exchange moves to mimic the time it would take to perform all of the necessary MD steps. While this artificial simulation allows us to investigate the potential strength of the method to tackle extremely rare events, it cannot reveal the effect of correlations between accepted paths when fast exploration of the reaction coordinate’s orthogonal directions is crucial. To analyze this effect, we also ran a two-dimensional (2D) membrane permeation system with two slightly asymmetric channels.^31^ Finally, to study our algorithm with a more generic *W*-matrix that needs to be solved via the BBFG formula, we also ran a set of underdamped Langevin simulations of a particle in a double-well potential^32^ using the recent wire fencing algorithm with the high-acceptance protocol.^28^ All simulation results were performed using five independent runs of 12 h. Errors were based on the standard deviations from these five simulations, except for the MSVS process, where a more reliable statistical error was desired for the comparison with analytical results. Here, block errors were determined on each of the five simulations based on the running average of the overall crossing probability. The block errors were finally combined to obtain the statistical error in the average of the five simulations.

### 4.1 Memoryless Single Variable Stochastic (MSVS) Process

**Figure 1:**
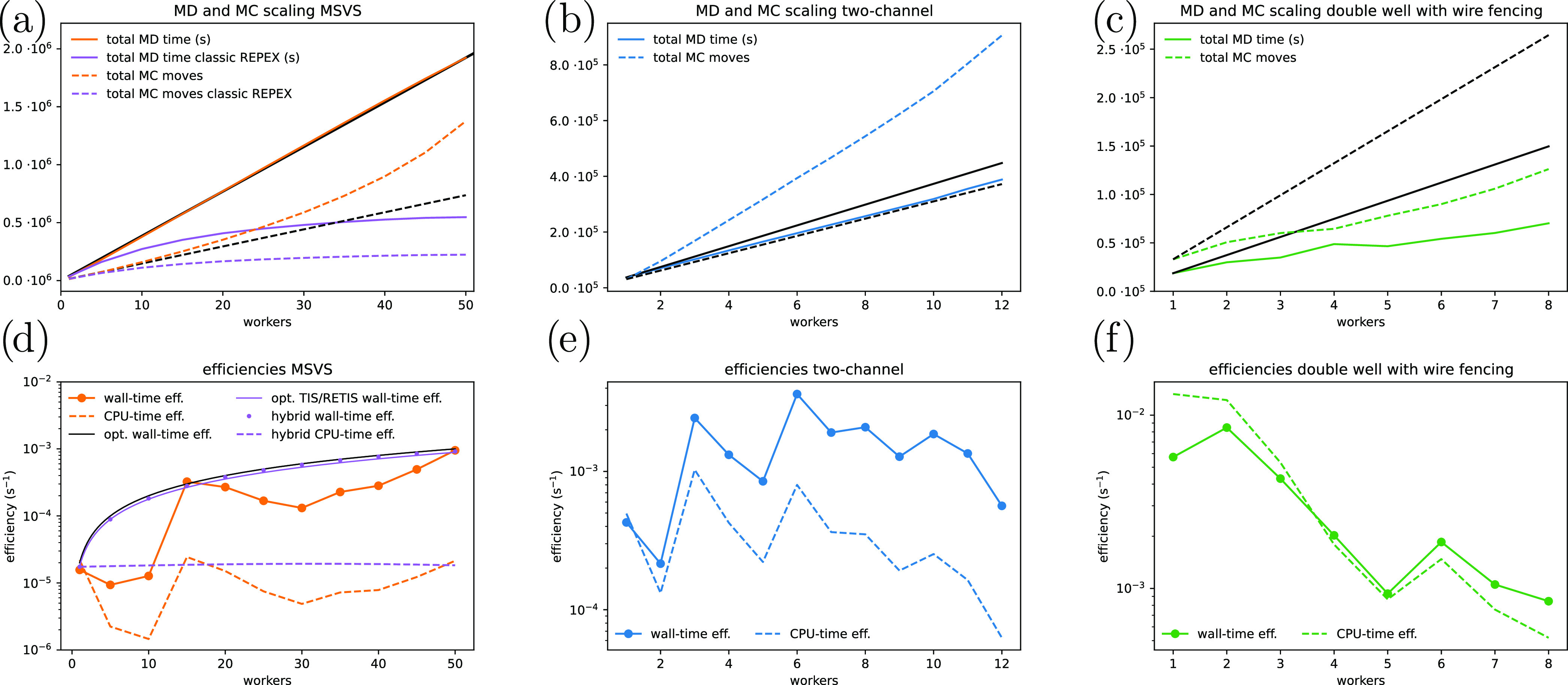
Average scaling of total MD time (cumulative time spent by all of the workers) (solid) and MC moves (dashed) (a–c) and wall-time (solid) and the CPU-time (dashed) efficiencies (d–f) for each number of workers. This is shown for the memoryless single variable stochastic (MSVS) process (a, d, orange), the two-channel system (b, e, blue), and the double well with wire fencing (c, f, green) simulations. Each of the data points is based on five independent simulations. For the scaling plots, the black lines are guides for linear scaling from the 1 worker data-point. The purple lines in the scaling plot for the MSVS simulations (a) show what the scaling would be if we had to wait for the slowest ensemble to finish for each MC move. The black line, purple line, purple dashed line, and points in the efficiency plot of the MSVS process (d) show the optimal, optimal TIS/RETIS, hybrid CPU-time efficiency, and hybrid wall-time efficiency, respectively, as computed in the SI.

Table 1 reports the overall crossing probabilities and their statistical errors for a system with 50 interfaces and 1, 5, 10, 15, 20, 25, 30, 35, 40, 45, and 50 workers. All values are within a 50% deviation from the true value of 10^–50^ with the more accurate estimates for the simulations having a large number of workers. Also, the true value is within one standard deviation of the reported averages for 70% of the data points, as is expected from the standard Gaussian confidence intervals. Figure 1a shows the scaling of the MD time (solid lines) and number of MC moves (dashed lines) of the MSVS simulations (orange) compared to linear scaling (black) and the expected scaling for standard replica exchange (REPEX) in which ensembles are updated in cohort (purple).

**Table 1:** Results of the Three Model Systems Showing Crossing Probabilities (*P*_cross_), Permeabilities (perm.), and Rates for Different Number of Workers (*K*)^a^

MSVS		two-channel system			double well with wire fencing		
*K*	*P*_cross_/10^–50^	*K*	*P*_cross_/ 10^–5^	perm./10^–6^	*K*	*P*_cross_/10^–7^	rate/10^–7^
1	0.61 ± 0.33	1	1.52 ± 0.17	1.28 ± 0.14	1	5.91 ± 0.18	2.59 ± 0.07
5	1.47 ± 1.04	2	1.63 ± 0.24	1.37 ± 0.20	2	5.70 ± 0.13	2.51 ± 0.06
10	0.86 ± 0.51	3	1.52 ± 0.07	1.28 ± 0.06	3	5.57 ± 0.19	2.45 ± 0.08
15	0.68 ± 0.08	4	1.42 ± 0.10	1.19 ± 0.08	4	5.20 ± 0.30	2.34 ± 0.12
20	1.02 ± 0.13	5	1.40 ± 0.12	1.18 ± 0.10	5	5.05 ± 0.41	2.23 ± 0.18
25	1.02 ± 0.17	6	1.54 ± 0.06	1.30 ± 0.05	6	5.49 ± 0.29	2.42 ± 0.13
30	1.26 ± 0.24	7	1.48 ± 0.08	1.24 ± 0.07	7	4.99 ± 0.39	2.21 ± 0.17
35	1.05 ± 0.15	8	1.46 ± 0.08	1.23 ± 0.06	8	4.88 ± 0.43	2.15 ± 0.19
40	1.05 ± 0.14	9	1.42 ± 0.10	1.20 ± 0.08			
45	0.93 ± 0.09	10	1.44 ± 0.08	1.21 ± 0.07			
50	1.00 ± 0.07	11	1.41 ± 0.09	1.19 ± 0.08			
		12	1.30 ± 0.15	1.09 ± 0.12			
Literature/Theoretical Result							
			1.23 ± 0.16^b^	1.06 ± 0.14^b^			2.79 ± 0.70^d^
						5.84 ± 0.13^e^	2.58 ± 0.06^e^
	1.00^a^		1.61^c^	1.37^c^		5.83^c^	2.58^c^

a All results are shown in dimensionless units. Errors are based on single standard deviations. Values shown in the lower part are a: exact result, b: ref (31), c: approximated value based on Kramers’ theory (see the SI), d: ref (32), and e: ref (28).

Although the number of “MD steps” and MC moves quickly levels off to a nearly flat plateau in the standard approach due to workers being idle as they need to wait for the slowest worker, the replica exchange approach developed in this article shows a perfect linear scaling with respect to the MD time. The number of MC moves in the new method shows an even better than linear scaling due to the fact that the ensembles with shorter “path lengths” get simulated relatively more often with more workers, resulting in more MC moves per second. This in itself does not necessarily mean that the simulations converge much faster because the additional computational effort may not be targeted to the sampling where it is needed. If we neglect the fact that path ensemble simulations are correlated via the replica exchange moves, we can write that the relative error in overall crossing probability ϵ follows from the relative errors in each path ensemble ϵ_*i*_ via: ϵ^2^ = ∑_*i*_ ϵ_*i*_^2^. It is henceforth clear that additional computational power should not aim to lower the error in a few path ensembles that were already low compared to other path ensembles. We therefore measure the effectiveness of the additional workers by calculating computational efficiencies. The efficiency of a specific computational method is here defined as the inverse computer time, CPU- or wall-time, to obtain an overall relative error equal to 1: ϵ = 1.

In Figure 1d, the efficiencies based on wall-time (solid) and CPU-time (dashed) are plotted for the MSVS process. These plots depend on the ability of computing reliable statistical errors in the overall crossing probability that is an extremely small number, 10^–50^. The somewhat fluctuating behavior of these curves should hence be viewed as statistical noise as the confidence interval of these efficiencies depends on the statistical error of this error. Despite that, clear trends can be observed in which the CPU-time efficiency is more or less constant within statistical fluctuations, while the wall-time efficiency shows an upward trend. If we neglect the effect of replica exchange moves on the efficiency, we can relate these numerical results with theoretical ones^22,33^ for any possible division of a fixed total CPU-time over the different ensembles. A common sense approach would be to aim for the same error ϵ_*i*_ in each ensemble (which implies doing the same number of MC moves per ensemble) or to divide the total CPU-time evenly over the ensembles. These two strategies correspond to the case *K* = 1 or standard RETIS and *K* = *N* or standard TIS, respectively. Ref (33) showed that these two strategies provide the same efficiency, and in the SI, we derive that this leads to a wall-time efficiency as a function of the number of workers (*K*) equal to *K*/56250, which is denoted by the continuous purple line in Figure 1d (the hypothetical wall-time efficiency for a parallel simulation that uses the CPU hours equally efficient as TIS and serial RETIS, see eq 50 in the SI). The optimum division, however, would give a slightly better wall-time efficiency equal to *K*/50,000 which is the continuous black line in this figure. Also shown in Figure 1d are the expected theoretical efficiencies based on the numerical distribution of MC moves in each ensemble. This hybrid numerical/theoretical result is shown by the small purple dots and the dashed purple line. This shows that ∞RETIS, at least for a system in which the path length grows linearly with the ensemble’s rank, naturally provides a division of the computational resources that is even better than TIS (*K* = *N*) or RETIS (*K* = 1). Yet, due to statistical inaccuracies this is only evident for the *K* = 15 case if we base our analysis on the numerical block errors.

In any case, it shows that the possible concern that additional CPU resources in parallel ∞RETIS runs may not be properly targeted due to oversampling of the ensembles with the shorter paths is unfounded. On the contrary, even the CPU efficiency seems to improve slightly compared to TIS and RETIS that have the same CPU efficiency. This is maybe not so surprising since the division of CPU hours over the different ensembles in ∞RETIS is somewhat in between the divisions what one gets with TIS and RETIS, and the optimum^33^ is also in between TIS and RETIS. In addition, we leave the number of interfaces and their locations unchanged in our analysis. However, the flexibility of ∞RETIS makes it very easy to add additional interfaces. By placing a higher density of interfaces high on the barrier, it is also possible to target more CPU to the expensive ensembles. This higher density has the additional benefit that the local crossing probabilities in this area are increased so that fewer paths are needed to calculate them.

Coming back to Figure 1d, we see that the best wall-time efficiency is obtained for the case *K* = *N*, which is essentially equivalent of running independent TIS simulations (i.e., without doing any replica exchange moves). We do not expect this to apply to more complex systems where the replica exchange move is a proven weapon for efficient sampling.

### 4.2 Two-Channel Simulations

In the middle column of Table 1, we report the calculated crossing probabilities and permeabilities for five simulations for every number of workers. All simulations are somewhat higher, though still in good agreement with the previous simulation from ref (31). We also evaluated the approximate result based on Kramers’ theory (see the SI), which seems to confirm the results obtained in this paper. Figure 1b shows the scaling of the MD time (solid lines) and number of MC moves (dashed lines) of the two-channel simulations (blue) compared to linear scaling (black). We see a slightly worse than linear scaling of the MD time, which might just be due to a small positive fluctuation of the 1 worker data-point. We also see a similar more than linear scaling in the number of MC moves as with the MSVS simulations, for the same reason. In Figure 1e, the efficiencies based on wall-time (solid) and CPU-time (dashed) are plotted for the two-channel system. The CPU-time efficiency is more or less flat until 8 workers after which it starts to drop off. The wall-time efficiency shows an upward trend until 10 workers after which it starts to drop off as well. We assign this drop to the reduction of replica exchange moves which is an essential aspect for sampling this system efficiently.^31^ This is tangible from Figure S1 in the SI where we plot fraction of trajectories, passing through λ_*M*–1_, that are in the lower barrier channel. While from the average fraction it still looks like the simulations sampled both channels for any number of workers, 4 out of the 5 simulations in the *K* = *N* = 12 case solely visited one of the two channels. This is in agreement with previous TIS results.^31^ The *K* = 11 case already provides a dramatic improvement, but is still expected to be suboptimal due to the relatively low frequency of replica exchange moves compared to *K* < 11. From this 2D system, it would indicate that having *K* ≈ *N*/2 is a safe bet for optimum efficiency.

### 4.3 Double-Well 1D Barrier Using Wire Fencing

In the right column of Table 1, we report the calculated crossing probabilities and rates for the underdamped Langevin particle in the 1D double-well potential. All simulations are in reasonable agreement with each other and the results of refs (28) and (32), as well as the approximate value based on Kramers’ theory. However, while these results confirm the soundness of the method, the scaling and efficiency are less convincing. Figure 1c shows a significantly worse than linear scaling. On further inspection, we found the average time per MC move was significantly smaller than our infinite swapping time (1 s) when the simulation was run with more than two workers. This results in a bottleneck on how many MC moves can be started per second, which is the reason for the observed bad scaling. It still is slightly positive instead of flat as the infinite swapping procedure becomes quicker with more workers due to the smaller *W*-matrix. The same bottleneck can be seen in Figure 1f where both efficiencies plummet with more than two workers. The reported scaling deficiency is of little significance for actual molecular systems where the creation of a full path takes minutes to hours rather than subseconds.

## 5 Conclusions

We developed a new generic replica exchange method that is able to effectively deal with MC moves with varying CPU costs, for instance, due to the algorithmic complexity of the MC moves. An essential aspect of the method is that the number of workers, who execute the ensemble’s specific MC moves in parallel, is less than the number of ensembles. Once a worker is finished with its move, replica exchange moves are carried out solely between those ensembles that are not occupied by a worker. This implies that the ensembles are updated at irregular intervals and a different number of MC moves will be executed for each ensemble. As a result, the conceptual viewpoint in which the set of replicas are viewed as a single superstate is no longer valid and the existence of some kind of detailed-balance relation is no longer trivial. To prove the exactness of our approach, we introduced new conceptual views on the replica exchange methodology that is different from the common superstate principle. Instead, we show that the distributions in the new approach are conserved for each ensemble individually via a twisted detailed-balance relation in which the other ensembles constitute an environment that is potentially actively involved in the MC move of the considered ensemble. In addition, the method can be combined with an infinite swapping approach without factorial scaling based on a mathematical reformulation using permanents.

We applied the novel replica exchange technique on a path sampling algorithm, RETIS, which is a prototype of algorithm where the costs for a Monte Carlo move can vary enormously. The resulting new path sampling algorithm, coined ∞RETIS, was thereafter tested on three model systems. The results of these simulations show that the number of MD steps increases linearly with the number of workers invoked as long as the ensemble’s MC move has a lower computational cost than the replica exchange move carried out by the scheduler. The number of executed MC moves shows an even better than linear scaling. Moreover, the efficiency increases linearly with the number of workers for a low-dimensional system in which the replica exchange move has little effect, while it has an optimum in more complex systems as the number of successful replica exchange moves decreases when the number of workers is close to the number of ensembles.

In summary, the replica exchange method discussed in this paper has a clear potential to accelerate present path sampling simulations, but can also be combined with many other complex algorithms including those that are yet to be invented. With the continuing trend to run progressively more massively parallel computing jobs, our algorithm is likely to gain importance. In the case of ∞RETIS, we envision many applications in the fields of nucleation, self-assembly, chemical reactions, enzymatic catalysis, membrane permeation, protein folding, and other conformational changes in biomolecules. The ∞RETIS method and the noncohort replica exchange method, in general, are therefore expected to open up new avenues in the field of molecular simulations and maybe even beyond.

## Supporting Information Available

Details of the simulations, proof that the replica exchange method with cost unbalanced replicas conserves the equilibrium distribution at the individual ensemble level;  algorithm for computing the *P*-matrix from a *W*-matrix for the case that the *W*-matrix consists of rows having a series with ones, followed by zeros; derivations of the theoretical results on the crossing probabilities, rate constant and permeability via Kramers’ theory that are shown in Table 1; discussion on the computational efficiencies with the derivations for the most optimal efficiencies; some additional simulation results on the relative transition probabilities through the lower and higher barrier channel for the two-channel system; our current code with which the model system calculations were done and the project started for advanced simulations (PDF)
