# Supplementary material for: Exchanging Replicas with Unequal Cost, Infinitely and Permanently
Source: J Phys Chem A. 2022 Nov 17;126(47):8878–86. doi: 10.1021/acs.jpca.2c06004 (PMC9720720; doi:10.1021/acs.jpca.2c06004)
Supplement: Supplementary file 1 — jp2c06004_si_001.pdf [file jp2c06004_si_001.pdf]

# Supporting Information for: Exchanging Replicas with Unequal Cost, Infinitely and Permanently

Sander Roet[a], Daniel T. Zhang[a], and Titus S. van Erp[a*]

[a]*Department of Chemistry, Norwegian University of Science and Technology (NTNU), N-7491 Trondheim, Norway*

E-mail: titus.van.erp@ntnu.no

## 1 Supporting Information Text

This Supplementary Information contains the following data, derivations, and numerical examples. In Sec. 2, we describe the complete implementation details for all the ∞RETIS simulations. In Sec. 3, we provide a proof that the replica exchange method with cost unbalanced replicas conserves the equilibrium distribution at the individual ensemble level. Instead of the superstate principle, the derivation is based on the individual ensemble's perspective where the other ensembles serve as an environment, which finally leads to a twisted detailed-balance relation. In Sec.4, we show a $\mathcal{O}(n^2)$ algorithm for computing the $P$-matrix from a $W$-matrix for the case that the $W$-matrix consists of rows having a series with ones, followed by zeros. This is the type of matrix that is relevant for RETIS simulations based on the standard shooting move. Sec. 5 presents the derivations of the theoretical results on the crossing probabilities, rate constant, and permeability via Kramers' theory that are shown in table 1 of the main article. In Sec. 6 the computational efficiencies, including the

derivations for the most optimal efficiencies, are discussed. Finally, in Sec. 7 we provide some additional simulation results on the relative transition probabilities through the lower and higher barrier channel.

## 2 Simulation Methods

The implementation of ∞RETIS was structured as follows. We start $1 \leq K \leq N$ worker- and 1 scheduler-process. Each of the worker-processes is going to process ensemble specific MC moves while the scheduler-process will do all the replica exchange moves and submits new jobs to the workers. All ensembles/trajectories that are currently being updated by a worker-process are not considered for MC moves by the scheduler, essentially being 'locked'. This means that no data is written for those ensembles and they are not valid targets for swapping moves. After a worker is done, it submits the result to the scheduler, the scheduler then unlocks the returned ensemble/trajectory and executes the replica exchange moves on all ensembles/trajectories that that are not locked. It then submits a new job to the freed worker for performing a new MC move in a randomly chosen free ensemble (or two ensembles in case of a point exchange move) and locks the involved ensembles/trajectories.

In the ∞RETIS method there are two kind of ensemble moves that involve MD steps. The first one is the shooting move (either standard shooting[1] or the more recent sub-trajectory moves[2,3]) in which a new path is being generated from an old path within a single ensemble. The second one is the point exchange move between $[0^-]$ and $[0^+]$. If a worker is assigned to this task, it means that both $[0^-]$ and $[0^+]$ are occupied by this worker. The scheduler ensures that there is never more than 1 worker considered free at a given time. When the free worker is assigned to perform a new MC move, each of the ensembles have an equal probability to be selected. If $[0^+]$ or $[0^-]$ is selected and the other is also free, there is a 50% chance to perform a $[0^-] \leftrightarrow [0^+]$ point exchange move instead of a shooting move in the selected ensemble.

## Memoryless single variable stochastic (MSVS) process

No actual MD is run for the MSVS simulations. Instead, we directly sample two random numbers, $r_1$ and $r_2$ from an uniform distribution $\in [0, 1)$ to set the path's progress and the path length. A path in ensemble $[k^+]$ is assumed to cross interface $\lambda_{k+l}$ if $r_1 < (0.1)^l$. After this, we wait a random time, $t = 0.2\, r_2\, k + 0.1$ in seconds. This was done to simulate both the increasing average simulation time and variance for outer ensembles. This setup means that we have no history dependence and allows us to compute the theoretical values shown in figure 1. 5 independent ∞RETIS simulations were run with $1, 5, 10, 15, \ldots, 45, 50$ workers.

## Two-channel simulations

In order to investigate the effect of our algorithm on the ergodicity of the sampling, a 2D two-channel simulation was run as described in reference[4]. The new RETIS moves introduced in that paper (mirror-move and target-swap move) were not used. Instead, MD was only run to do shooting moves or the $[0^-] \leftrightarrow [0^+]$ point exchanges. As the MD for this system completed too fast, every worker was set to wait 9 times the time it took to run the MD before returning the result. 5 independent ∞RETIS simulations were run with $1, 2, \ldots, 11, 12$ workers.

## 1D double well with wire fencing

In order to investigate the accuracy with a $W$ matrix that contains more numbers than 0s or 1s we simulated a 1D double-well system[5] together with the high-acceptance version of a novel path-sampling algorithm, wire fencing. The algorithm is described in reference[3], but for us the relevant part is that the high-acceptance weight is the number of frames that a path has outside the interface for each ensemble times an extra factor 2 if the path ends at the last interface. As for the two-channel system, a worker was set to wait 9 times the time it took to complete the MD move before returning the result. 5 independent ∞RETIS simulations were run with $1, 2, \ldots, 7, 8$ workers with interfaces placed at

$[-0.99, -0.8, -0.7, -0.6, -0.5, -0.4, -0.3, 1.0]$.

# 3 Detailed-balance relations

In this section, we will derive detailed-balance relations for parallel replica's that are not based on the common superstate viewpoint. These alternative relations can be used to validate the replica exchange algorithm for replica's with unequal CPU cost. Our derivation is based on the finite swapping approach, though the infinite swapping version follows automatically from this when the probability to perform a swap goes to unity ($P_{\mathrm{RE}} \to 1$) as explained in the main text. To simplify matters, we assume that we have one type of replica exchange move that is low in CPU cost and one type of ensemble move that operates within one ensemble and has a high CPU cost. The relations that we derive are, however, by no means limited to that. In fact, in the RETIS algorithm there is also a point exchange move between the $[0^-]$ and $[0^+]$ ensemble. In previous publications this move, annotated as $[0^-] \leftrightarrow [0^+]$, was categorized as a special type of swapping/replica exchange move. In this article we reserve the name swap or replica exchange to an operation that involves the swapping of full paths, which does not require any MD steps. In contrast, the $[0^-] \leftrightarrow [0^+]$ point exchange implies the exchange of time slices at the end and start of the paths that are then extended at the other side of the $\lambda_0$ interface. In our implementation, this $[0^-] \leftrightarrow [0^+]$ move is carried out by a single worker that locks both the $[0^-]$ and $[0^+]$ ensembles during this move. As the $[0^-]$ paths can never be swapped with any of the other paths, we can view the point exchange move as an ensemble move in ensemble $[0^+]$.

As explained in the main article, the replica exchange algorithm that we propose is based on a set of workers and a set of ensembles. The number of workers $K$ is less than the number of ensembles $N$. Most of the time the worker is performing a CPU intensive single-ensemble move. The ensemble in which the worker operates is considered occupied/locked. Once a worker has completed a CPU intensive move, the move will be accepted or rejected,

after which either a replica exchange move will be carried out with any of the unoccupied ensembles or the worker will be assigned to do a new single-ensemble move at a randomly picked free ensemble.

In order to indicate the difference between occupied and unoccupied ensembles, we introduce a new state vector that indicates both the available ensembles as in the main text and the occupied ensembles with a bar, e. g: $S = (s_1, s_2, \overline{s_3}, s_4, \overline{s_5})$ to show that there are 5 ensembles of which ensemble 3 and 5 are occupied by a worker. For both occupied and unoccupied ensembles, the $s_i$-terms reflect the most recent state that was sampled in the $i$th ensemble. Now our sole aim is to ensure that if we just count the instances that an ensemble $i$ is updated with a new sample (which could be a copy of the previous sample in case of a rejected move), these should be distributed according the correct probability density $\rho_i$.

It is important to note that the time between two updates can vary and depends on the state that was most recently sampled. However, the waiting time between an update of a specific ensemble and the point in time that this ensemble gets occupied by a worker will depend on the states of all other ensembles, but *not* on the state in the ensemble considered. Since the ensembles are independent, this waiting time will be the same on average irrespective to this sampled state. This has as a consequence that if we take "photographs" of the state vector, at intervals or randomly, evenly distributed over time, we should again obtain the correct distributions $\rho_i$, for all $i$, of the states in ensemble $i$ as long as we ignore the instances that this ensemble is occupied. In other words, we can write for the previous example state vector

$$\rho(S) = \rho(s_1, s_2, \overline{s_3}, s_4, \overline{s_5}) = \rho_1(s_1)\rho_2(s_2)\rho_3^u(\overline{s_3})\rho_4(s_4)\rho_5^u(\overline{s_5}) \tag{1}$$

where $\rho_i(\cdot)$ is the statistically correct distribution of ensemble $i$, and $\rho_j^u(\cdot)$ an unknown distribution for occupied ensemble $j$ that has no clear physical interpretation. For instance, it can happen that a state $s$ is relatively unlikely to exist in ensemble $i$, low $\rho_i(s)$, but that

any MC move starting from that state takes a very long time, resulting in a high $\rho_i^u(s)$.

Now, let's consider the Markov chain from the perspective of ensemble 1 where we monitor its state at the point that a new MC is initiated from an old state $s_1$. From the viewpoint of ensemble 1, the other ensembles are viewed as an "environment" ($\mathcal{E} = (s_2, \overline{s_3}, s_4, \overline{s_5})$ in the aforementioned example), that might or might not influence the MC move. The probability of state $s_1$ in ensemble 1 can be written as an integral of the conditional probability given an environment:

$$\rho_1(s_1) = \int \rho_1(s_1|\mathcal{E})\rho(\mathcal{E})\mathrm{d}\mathcal{E}. \tag{2}$$

As the ensembles are independent we can write

$$\rho_1(s_1|\mathcal{E}) = \rho_1(s_1), \tag{3}$$

but we temporary keep the condition to clarify the logical structure of the upcoming derivation.

As stated, we assume that we employ two types of moves: 1) a CPU intensive move that modifies $s_1$ without using the environment $\mathcal{E}$ and 2) a swapping move. In addition, the environment might influence the relative selection probabilities for choosing either 1) or 2). Typically, this selection probability will depend on $N_a(\mathcal{E})$, the number of unoccupied ensembles in $\mathcal{E}$. Further, we need to keep in mind that during the execution of the MC move in ensemble 1, the environment changes. How much the environment changes will depend on how long it takes to fully execute the move involving ensemble 1.

To derive detailed-balance relations for the replica exchange method for cost unbalanced ensembles, we start with the more general balance concept; if we have an infinite number of states distributed according to the equilibrium distribution, all of which make a MC move at the same time, then we have to get the equilibrium distribution again. This means that

the flux out off $s_1$ should be equal to the flux into $s_1$ which can be written as

$$\int \rho_1(s_1|\mathcal{E})\rho(\mathcal{E})\pi(s_1,\mathcal{E}\to s_1',\mathcal{E}')\mathrm{d}\mathcal{E}\mathrm{d}\mathcal{E}'\mathrm{d}s_1' = \int \rho_1(s_1''|\mathcal{E}'')\rho(\mathcal{E}'')\pi(s_1'',\mathcal{E}''\to s_1,\mathcal{E}''')\mathrm{d}\mathcal{E}''\mathrm{d}\mathcal{E}'''\mathrm{d}s_1'' \tag{4}$$

The transition probability $\pi(\cdot)$ can be split into the transitions via the different types moves (that we will indicate with the Greek letter $\alpha$) which will be selected with a probability $P_\alpha^{\mathrm{sel}}(\mathcal{E})$ that can depend on the environment $\mathcal{E}$:

$$\pi(s_1,\mathcal{E}\to s_1',\mathcal{E}') = \sum_\alpha P_\alpha^{\mathrm{sel}}(\mathcal{E})\pi_\alpha(s_1,\mathcal{E}\to s_1',\mathcal{E}') \tag{5}$$

This shows another complicating factor as in standard detailed-balance we need to consider the probability that the exact reverse move will be executed once the new state has been established. However, as the environment could have changed, the reverse move might involve different selection probabilities.

By substituting Eq. 5 into Eq. 4, we get an extra summation over $\alpha$ in addition to the integrals:

$$\begin{aligned}&\sum_\alpha \int \rho_1(s_1|\mathcal{E})\rho(\mathcal{E})P_\alpha^{\mathrm{sel}}(\mathcal{E})\pi_\alpha(s_1,\mathcal{E}\to s_1',\mathcal{E}')\mathrm{d}\mathcal{E}\mathrm{d}\mathcal{E}'\mathrm{d}s_1' = \\ &\sum_\alpha \int \rho_1(s_1''|\mathcal{E}'')\rho(\mathcal{E}'')P_\alpha^{\mathrm{sel}}(\mathcal{E}'')\pi_\alpha(s_1'',\mathcal{E}''\to s_1,\mathcal{E}''')\mathrm{d}\mathcal{E}''\mathrm{d}\mathcal{E}'''\mathrm{d}s_1''\end{aligned} \tag{6}$$

But at this point, we apply the first level of "detailedness" by requiring the equation to hold for *each* $\alpha$:

$$\begin{aligned}&\int \rho_1(s_1|\mathcal{E})\rho(\mathcal{E})P_\alpha^{\mathrm{sel}}(\mathcal{E})\pi_\alpha(s_1,\mathcal{E}\to s_1',\mathcal{E}')\mathrm{d}\mathcal{E}\mathrm{d}\mathcal{E}'\mathrm{d}s_1' = \\ &\int \rho_1(s_1''|\mathcal{E}'')\rho(\mathcal{E}'')P_\alpha^{\mathrm{sel}}(\mathcal{E}'')\pi_\alpha(s_1'',\mathcal{E}''\to s_1,\mathcal{E}''')\mathrm{d}\mathcal{E}''\mathrm{d}\mathcal{E}'''\mathrm{d}s_1''\end{aligned} \tag{7}$$

So now we can evaluate the different moves separately. We further simplify this expression

by integration out the variables $\mathcal{E}'$ and $\mathcal{E}'''$ using the following relation:

$$\int \pi_\alpha(s, \mathcal{E} \rightarrow s', \mathcal{E}')\mathrm{d}\mathcal{E}' = \pi_\alpha(s, \mathcal{E} \rightarrow s', {}^a\mathcal{E}) \tag{8}$$

where ${}^a\mathcal{E}$ refers to *any* possible environment. Substitution of Eq. 8 in Eq. 7 gives:

$$\int \rho_1(s_1|\mathcal{E})\rho(\mathcal{E})P_\alpha^{\mathrm{sel}}(\mathcal{E})\pi_\alpha(s_1, \mathcal{E} \rightarrow s_1', {}^a\mathcal{E})\mathrm{d}\mathcal{E}\mathrm{d}s_1' = \int \rho_1(s_1''|\mathcal{E}'')\rho(\mathcal{E}'')P_\alpha^{\mathrm{sel}}(\mathcal{E}'')\pi_\alpha(s_1'', \mathcal{E}'' \rightarrow s_1, {}^a\mathcal{E})\mathrm{d}\mathcal{E}''\mathrm{d}s_1'' \tag{9}$$

First, we consider $\alpha = 1$ referring the CPU intensive move that only operates in ensemble 1. For this move we substitute $\alpha = 1$ in Eq. 9 and replace $\mathcal{E}''$ and $s_1''$ with respectively $\mathcal{E}$ and $s_1'$, which is allowed since these are dummy integration variables

$$\int \rho_1(s_1|\mathcal{E})\rho(\mathcal{E})P_1^{\mathrm{sel}}(\mathcal{E})\pi_1(s_1, \mathcal{E} \rightarrow s_1', {}^a\mathcal{E})\mathrm{d}\mathcal{E}\mathrm{d}s_1' = \int \rho_1(s_1'|\mathcal{E})\rho(\mathcal{E})P_1^{\mathrm{sel}}(\mathcal{E})\pi_1(s_1', \mathcal{E} \rightarrow s_1, {}^a\mathcal{E})\mathrm{d}\mathcal{E}\mathrm{d}s_1'$$

Then, we fix another level of detailedness by requiring that the integrands at the left and right side of equality sign to be identical for any $\mathcal{E}$ and $s_1'$. As a result, $\rho(\mathcal{E})P_\alpha^{\mathrm{sel}}(\mathcal{E})$ will cancel out such that we can write

$$\rho(s_1|\mathcal{E})\pi_1(s_1, \mathcal{E} \rightarrow s_1', {}^a\mathcal{E}) = \rho(s_1'|\mathcal{E})\pi_1(s_1', \mathcal{E} \rightarrow s_1, {}^a\mathcal{E}) \tag{10}$$

Since in move 1) the ensembles progress independently from each other, we have

$$\pi_1(s_1, \mathcal{E} \rightarrow s_1', {}^a\mathcal{E}) = \pi_1(s_1 \rightarrow s_1')\pi_1(\mathcal{E} \rightarrow {}^a\mathcal{E}) \tag{11}$$

The subscript "1" in $\pi_1(\mathcal{E} \rightarrow {}^a\mathcal{E})$ might seem contradictory to the previous statement on independent progression, but it just indicates that the points in time at which the environment is evaluated relates the duration of the MC move in ensemble 1: $\mathcal{E}$ is the environment at the start of the MC move in ensemble 1, and ${}^a\mathcal{E}$ is that when the move is completed. As

the time for a $s_1 \to s_1'$ move is likely not the same as the time for a $s_1' \to s_1$ move, the final environments are likely not the same. However, ${}^{a}\mathcal{E}$ refers to *any* environment. Hence, by substituting Eq. 11 into Eq. 10, $\pi_1(\mathcal{E} \to {}^{a}\mathcal{E})$ does not only cancel as it appears at both sides of the equals sign, it is also equal to one. We therefore have not just one, but two very good reasons to eliminate this term such that:

$$\rho_1(s_1|\mathcal{E})\pi_1(s_1 \to s_1') = \rho_1(s_1'|\mathcal{E})\pi_1(s_1' \to s_1) \tag{12}$$

or, via Eq. 3:

$$\rho_1(s_1)\pi_1(s_1 \to s_1') = \rho_1(s_1')\pi_1(s_1' \to s_1) \tag{13}$$

This equation essentially the same as the standard detailed balance equation such that we can adapt our acceptance according to

$$P_{\mathrm{acc}}(s_1 \to s_1') = \min\left[1, \frac{\rho_1(s_1')P_{\mathrm{gen}}(s_1' \to s_1)}{\rho_1(s_1)P_{\mathrm{gen}}(s_1 \to s_1')}\right] \tag{14}$$

which is exactly the same as in standard Metropolis-Hastings. Still, the underlying philosophy is different from a super-state perspective as the number of transitions from old to new, $S^{(o)} \to S^{(n)}$, is not the same as from new to old, $S^{(n)} \to S^{(o)}$. Instead, by writing $S = (s_1, \mathcal{E})$ we have that the number of $(s_1^{(o)}, \mathcal{E}^{(o)}) \to (s_1^{(n)}, {}^{a}\mathcal{E}^{(n)})$ transitions should be equal to the number of $(s_1^{(n)}, \mathcal{E}^{(o)}) \to (s_1^{(o)}, {}^{a}\mathcal{E}^{(n)})$ transitions. In addition, as at the end of the move we only update ensemble 1, and not those that are here considered as environment, the number of sampled states in the ensembles do not increase in cohort. Sampling all states simultaneously like in a true superstate move would imply that distributions get mixed with the unknown and unphysical $\rho_i^u$ distributions.

For the swapping move we just consider the example of an attempted $1 \leftrightarrow 2$ swap as all other swaps $i \leftrightarrow j$ are completely analogous. We start again at Eq. 7 with $\alpha = 1 \leftrightarrow 2$, and

further we split the environment $\mathcal{E} = \{s_2, \mathcal{E}_{\not 2}\}$ into the part that participates in the swap move, $s_2$, and the rest, $\mathcal{E}_{\not 2}$:

$$\int \rho_1(s_1|s_2,\mathcal{E}_{\not 2})\rho_2(s_2)\rho(\mathcal{E}_{\not 2})P^{\text{sel}}_{1\leftrightarrow 2}(\mathcal{E}_{\not 2}) \times \pi_{1\leftrightarrow 2}(s_1, s_2, \mathcal{E}_{\not 2} \to s_1', s_2', \mathcal{E}_{\not 2}')\mathrm{d}s_2\mathrm{d}\mathcal{E}_{\not 2}\mathrm{d}s_2'\mathrm{d}\mathcal{E}_{\not 2}'\mathrm{d}s_1' =$$
$$\int \rho_1(s_1''|s_2'',\mathcal{E}_{\not 2}'')\rho_2(s_2'')\rho(\mathcal{E}_{\not 2}'')P^{\text{sel}}_{1\leftrightarrow 2}(\mathcal{E}_{\not 2}'') \times \pi_{1\leftrightarrow 2}(s_1'', s_2'', \mathcal{E}_{\not 2}'' \to s_1, s_2''', \mathcal{E}_{\not 2}''')\mathrm{d}s_2''\mathrm{d}\mathcal{E}_{\not 2}''\mathrm{d}s_2'''\mathrm{d}\mathcal{E}_{\not 2}'''\mathrm{d}s_1'' \tag{15}$$

Here, we assume that the selection probability $P^{\text{sel}}_{1\leftrightarrow 2}$ depends on $\mathcal{E}_{\not 2}$. The chance to do a replica exchange move equals $P_{\text{RE}}$, but once it is decided to perform a replica exchange move, all possible swaps $i \leftrightarrow j$ compete to be selected with an equal probability. Hence, the probability for the $1 \leftrightarrow 2$ swap to be selected depends on the number of available ensembles, which is the total number of ensembles minus the number of occupied ones. This latter information is contained in $\mathcal{E}_{\not 2}$

The swapping transition probability $\pi_{1\leftrightarrow 2}$ relates to a move that has only one possible outcome, namely the one in which the states in ensemble 1 and 2 are exchanged. Therefore, $\pi_{1\leftrightarrow 2}(s_1, s_2, \mathcal{E}_{\not 2} \to s_1', s_2', \mathcal{E}_{\not 2}')$ is vanishing if $s_1' \neq s_2$ and $s_2' \neq s_1$. Likewise, $\pi_{1\leftrightarrow 2}(s_1'', s_2'', \mathcal{E}_{\not 2}'' \to s_1, s_2''', \mathcal{E}_{\not 2}''')$ vanishes if $s_2'' \neq s_1$ and $s_1'' \neq s_2'''$. We can, therefore, write

$$\begin{aligned} \pi_{1\leftrightarrow 2}(s_1, s_2, \mathcal{E}_{\not 2} \to s_1', s_2', \mathcal{E}_{\not 2}') &= \hat{\pi}_{1\leftrightarrow 2}(s_1, s_2, \mathcal{E}_{\not 2} \to s_2, s_1, \mathcal{E}_{\not 2}')\delta(s_2 - s_1')\delta(s_1 - s_2') \\ \pi_{1\leftrightarrow 2}(s_1'', s_2'', \mathcal{E}_{\not 2}'' \to s_1, s_2''', \mathcal{E}_{\not 2}''') &= \hat{\pi}_{1\leftrightarrow 2}(s_2''', s_1, \mathcal{E}_{\not 2}'' \to s_1, s_2''', \mathcal{E}_{\not 2}''')\delta(s_2''' - s_1'')\delta(s_1 - s_2'') \end{aligned} \tag{16}$$

where the transition probability with the hat, $\hat{\pi}_{1\leftrightarrow 2}$, differs from transition probability without the hat, $\pi_{1\leftrightarrow 2}$, by the fact that the latter considers any potential (even if impossible) result of the swapping operation, while the former actually relates to the probability of successfully executing the move in practice in which $s_1$ and $s_2$ change places. Substitution of Eqs. 16 in Eq. 15 allows us to eliminate the integrals over $s_1'$, $s_2'$, $s_1''$, and $s_2''$ via the

delta-function integration property.

$$\int \rho_1(s_1|s_2,\mathcal{E}_{\not 2})\rho_2(s_2)\rho(\mathcal{E}_{\not 2})P^{\text{sel}}_{1\leftrightarrow 2}(\mathcal{E}_{\not 2})\hat{\pi}_{1\leftrightarrow 2}(s_1,s_2,\mathcal{E}_{\not 2}\rightarrow s_2,s_1,\mathcal{E}'_{\not 2})\mathrm{d}s_2\mathrm{d}\mathcal{E}_{\not 2}\mathrm{d}\mathcal{E}'_{\not 2}=$$
$$\int \rho_1(s_2'''|s_1,\mathcal{E}''_{\not 2})\rho_2(s_1)\rho(\mathcal{E}''_{\not 2})P^{\text{sel}}_{1\leftrightarrow 2}(\mathcal{E}''_{\not 2})\hat{\pi}_{1\leftrightarrow 2}(s_2''',s_1,\mathcal{E}''_{\not 2}\rightarrow s_1,s_2''',\mathcal{E}'''_{\not 2})\mathrm{d}\mathcal{E}''_{\not 2}\mathrm{d}s_2'''\mathrm{d}\mathcal{E}'''_{\not 2} \tag{17}$$

We then eliminate the integrals over $\mathcal{E}'_{\not 2}$ and $\mathcal{E}'''_{\not 2}$ using a similar expression as Eq. 8.

$$\int \rho_1(s_1|s_2,\mathcal{E}_{\not 2})\rho_2(s_2)\rho(\mathcal{E}_{\not 2})P^{\text{sel}}_{1\leftrightarrow 2}(\mathcal{E}_{\not 2})\hat{\pi}_{1\leftrightarrow 2}(s_1,s_2,\mathcal{E}_{\not 2}\rightarrow s_2,s_1,{}^{a}\mathcal{E}_{\not 2})\mathrm{d}s_2\mathrm{d}\mathcal{E}_{\not 2}=$$
$$\int \rho_1(s_2'''|s_1,\mathcal{E}''_{\not 2})\rho_2(s_1)\rho(\mathcal{E}''_{\not 2})P^{\text{sel}}_{1\leftrightarrow 2}(\mathcal{E}''_{\not 2})\hat{\pi}_{1\leftrightarrow 2}(s_2''',s_1,\mathcal{E}''_{\not 2}\rightarrow s_1,s_2''',{}^{a}\mathcal{E}_{\not 2})\mathrm{d}\mathcal{E}''_{\not 2}\mathrm{d}s_2''' \tag{18}$$

In the next step, we change some of the dummy integration variable names: $s_2'''$ to $s_2$ and $\mathcal{E}''_{\not 2}$ to $\mathcal{E}_{\not 2}$.

$$\int \rho_1(s_1|s_2,\mathcal{E}_{\not 2})\rho_2(s_2)\rho(\mathcal{E}_{\not 2})P^{\text{sel}}_{1\leftrightarrow 2}(\mathcal{E}_{\not 2})\hat{\pi}_{1\leftrightarrow 2}(s_1,s_2,\mathcal{E}_{\not 2}\rightarrow s_2,s_1,{}^{a}\mathcal{E}_{\not 2})\mathrm{d}s_2\mathrm{d}\mathcal{E}_{\not 2}=$$
$$\int \rho_1(s_2|s_1,\mathcal{E}_{\not 2})\rho_2(s_1)\rho(\mathcal{E}_{\not 2})P^{\text{sel}}_{1\leftrightarrow 2}(\mathcal{E}_{\not 2})\hat{\pi}_{1\leftrightarrow 2}(s_2,s_1,\mathcal{E}_{\not 2}\rightarrow s_1,s_2,{}^{a}\mathcal{E}_{\not 2})\mathrm{d}\mathcal{E}_{\not 2}\mathrm{d}s_2 \tag{19}$$

and use a detailed-balance principle by stating that the equality does not only hold when integrated, but is true for any pair $s_2,\mathcal{E}_{\not 2}$.

$$\rho_1(s_1|s_2,\mathcal{E}_{\not 2})\rho_2(s_2)\hat{\pi}_{1\leftrightarrow 2}(s_1,s_2,\mathcal{E}_{\not 2}\rightarrow s_2,s_1,{}^{a}\mathcal{E}_{\not 2})=\rho_1(s_2|s_1,\mathcal{E}_{\not 2})\rho_2(s_1)\hat{\pi}_{1\leftrightarrow 2}(s_2,s_1,\mathcal{E}_{\not 2}\rightarrow s_1,s_2,{}^{a}\mathcal{E}_{\not 2}) \tag{20}$$

We further simplify $\rho_1(s_1|s_2,\mathcal{E}_{\not 2})$ by $\rho_1(s_1)$ using Eq. 3, and split $\hat{\pi}_{1\leftrightarrow 2}(s_1,s_2,\mathcal{E}_{\not 2}\rightarrow s_2,s_1,{}^{a}\mathcal{E}_{\not 2})$ into $\hat{\pi}_{1\leftrightarrow 2}(s_1,s_2\rightarrow s_2,s_1)\times\pi_{1\leftrightarrow 2}(\mathcal{E}_{\not 2}\rightarrow {}^{a}\mathcal{E}_{\not 2})$ where the latter term cancels like before:

$$\rho_1(s_1)\rho_2(s_2)\hat{\pi}_{1\leftrightarrow 2}(s_1,s_2\rightarrow s_2,s_1)=\rho_1(s_2)\rho_2(s_1)\hat{\pi}_{1\leftrightarrow 2}(s_2,s_1\rightarrow s_1,s_2) \tag{21}$$

Since $\hat{\pi}_{1\leftrightarrow 2}(s_2,s_1\rightarrow s_1,s_2)$ is the transition probability from $(s_1,s_2)$ to $(s_2,s_1)$ in the first two

ensembles given that the $1 \leftrightarrow 2$ swap move was selected, and given that there are no other possible outcomes of this swap ($P_{\rm gen} = 1$), the transition probability equals the acceptance probability:

$$\rho_1(s_1)\rho_2(s_2)P_{\rm acc}(s_1, s_2 \to s_2, s_1) = \rho_1(s_2)\rho_2(s_1)P_{\rm acc}(s_2, s_1 \to s_1, s_2) \tag{22}$$

To satisfy this relation, Eq. (4) of the main article suffices.

$$P_{\rm acc} = \min\left[1, \frac{\rho_1(s_2)\rho_2(s_1)}{\rho_1(s_1)\rho_2(s_2)}\right] \tag{23}$$

So also here, the standard replica exchange acceptance rule applies. The main difference is that ensembles are not updated in cohort. After the $1 \leftrightarrow 2$ swap move we only update ensembles 1 and 2. Alternatively, after the $1 \leftrightarrow 2$ swap all other free ensembles will be updated as well with "null moves". In the example of Eq. 1 this would mean that besides, ensemble 1 and 2, also ensemble 4 would be updated. As the state in this ensemble is not changing in a $1 \leftrightarrow 2$ swap, this would imply recounting the existing $s_4$ state. Hence, this could be viewed as a superstate move, but then without the occupied states. Resampling $s_4$ is allowed as the chance for resampling is independent of the content of ensemble 4. However, the sampling of the ensembles 3 and 5 should, while occupied, at all cost be avoided since the time that ensembles 3 and 5 remain occupied can correlate with the values of $s_3$ and $s_5$, respectively.

Like in Eq. 14, the acceptance rule of Eq. 23 is based on a twisted detailed balance relation: we require that, given an equilibrium distribution, the number of $(s_1^{(o)}, s_2^{(o)}, \mathcal{E}_{\not 2}^{(o)}) \to (s_1^{(n)}, s_2^{(n)}, {}^a\mathcal{E}_{\not 2}^{(n)})$ transitions should be equal to the number of $(s_1^{(n)}, s_2^{(n)}, \mathcal{E}_{\not 2}^{(o)}) \to (s_1^{(o)}, s_2^{(o)}, {}^a\mathcal{E}_{\not 2}^{(n)})$ transitions, where $s_1^{(o)} = s_2^{(n)} = s_1$ and $s_2^{(o)} = s_1^{(n)} = s_2$. So in this section, we proved that standard acceptance-rejection rules can be applied in a parallel scheme in which replica exchange moves occur only between unoccupied ensembles, such that ensembles are not updated in cohort.

# 4 Matrices with consecutive ones and zeros

If the high-acceptance approach is not applied, $w_i(X) = 1$ in Eq. (6) of the main article and the $W$-matrix has rows consisting of a sequence of ones, followed a sequence of zeros. The $P$-matrix can then be determined from Eq. (7) of the main article which has an $\mathcal{O}(n^2)$ scaling. In this section we provide the proof of this equation.

Let $n_i$ be the number of ones in row $i$. The first step to order the rows with increasing order of $n_i$. For instance in the following $5 \times 5$ matrix

$$W = \begin{array}{c} \\ s_1 \\ s_2 \\ s_3 \\ s_4 \\ s_5 \end{array} \begin{array}{c} \begin{array}{ccccc} e_1 & e_2 & e_3 & e_4 & e_5 \end{array} \\ \begin{pmatrix} 1 & 1 & 0 & 0 & 0 \\ 1 & 1 & 1 & 1 & 0 \\ 1 & 1 & 1 & 0 & 0 \\ 1 & 1 & 1 & 1 & 0 \\ 1 & 1 & 1 & 1 & 1 \end{pmatrix} \end{array}$$

we see that $s_2$, originating from an MC move in ensemble $e_2$, is also valid for $e_3$ and $e_4$. State $s_3$ that was created in $e_3$ only reaches the minimal condition for that ensemble. In path sampling, where $s_2$ and $s_3$ are paths and $e_2$, $e_3$ and $e_4$ refer to path ensembles $[k^+]$, $[l^+]$ and $[m^+]$ with $m > l > k$, it would mean that path $s_3$ crosses $\lambda_l$, but not $\lambda_m$, while path $s_2$ crosses at least $m - k$ more additional interfaces than strictly needed for being a valid trajectory in $e_2 = [k^+]$. As a result, the third row has fewer ones than the second row. After

reordering, the $W$-matrix looks as follows:

$$W = \begin{array}{c} \\ s'_1 = s_1 \\ s'_2 = s_3 \\ s_3 = s_2 \\ s'_4 = s_4 \\ s'_5 = s_5 \end{array} \begin{array}{c} \begin{array}{ccccc} e_1 & e_2 & e_3 & e_4 & e_5 \end{array} \\ \begin{pmatrix} 1 & 1 & 0 & 0 & 0 \\ 1 & 1 & 1 & 0 & 0 \\ 1 & 1 & 1 & 1 & 0 \\ 1 & 1 & 1 & 1 & 0 \\ 1 & 1 & 1 & 1 & 1 \end{pmatrix} \end{array} = W[n_1, n_2, n_3, n_4, n_5] = W[2, 3, 4, 4, 5]$$

where we introduced the bracket notation $W[\cdot]$ indicating the number of ones in each row in which $1 \leq n_1 \leq n_2 \leq n_3 \ldots \leq n_n = n$. Likewise, we always have $n_i \geq i$.

Based on the recursive relation, $\mathrm{perm}(W) = \sum_j W_{1j}\mathrm{perm}(W\{1j\})$, and the fact that the matrix after removing row 1 and column $j$, $W\{1j\}$, is identical for any $j \leq n_1$, we can write

$$\mathrm{perm}(W[n_1, n_2, n_3, \ldots, n_n]) = n_1 \times \mathrm{perm}(W[n_2 - 1, n_3 - 1, \ldots, n_n - 1]) \tag{24}$$

The permanent of the remaining matrix $W[n_2 - 1, n_3 - 1, \ldots, n_n - 1]$ can again be written as $(n_2 - 1) \times \mathrm{perm}(W[n_3 - 2, \ldots, n_n - 2])$ and so on. The permanent is, hence, equal to

$$\mathrm{perm}(W[n_1, n_2, \ldots, n_n]) = \prod_{i=1}^{n} (n_i + 1 - i) \tag{25}$$

The $P$-matrix follows from Eq. (5) of the main article: $P_{ij} = W_{ij}\mathrm{perm}(W\{ij\})/\mathrm{perm}(W)$. This means that $P_{ij} = 0$ whenever $W_{ij} = 0$. If $W_{ij} = 1$, and $n_{i-1} < j$ or $i = 1$, we have that for a matrix $W[n_1, n_2, \ldots, n_{i-1}, n_i, n_{i+1}, \ldots, n_n]$ the following matrix remains after removal of row $i$ and column $j$:

$$W\{ij\} = W[n_1, n_2, \ldots, n_{i-1}, n_{i+1} - 1, \ldots, n_n - 1] \tag{26}$$

and the permanent

$$\begin{aligned}\mathrm{perm}(W\{ij\}) &= \left(\prod_{i'=1}^{i-1}(n_{i'}+1-i')\right)\left(\prod_{i'=i+1}^{n}(n_{i'}-1+1-(i'-1))\right) \\ &= \left(\prod_{i'=1}^{i-1}(n_{i'}+1-i')\right)\left(\prod_{i'=i+1}^{n}(n_{i'}+1-i')\right) = \frac{\mathrm{perm}(W)}{(n_i+1-i)} \end{aligned} \tag{27}$$

and, therefore, for this case we have

$$P_{ij} = \frac{1 \times \mathrm{perm}(W\{ij\})}{\mathrm{perm}(W)} = \frac{1}{(n_i+1-i)}. \tag{28}$$

If for some $k < i$, $n_k \geq j$, while $n_{k-1} < j$ or $k = 1$, we have that for a matrix $W[n_1, n_2, \ldots, n_{k-1}, n_k, \ldots, n_i, n_{i+1}, \ldots, n_n]$ the following matrix remains after removal of row $i$ and column $j$:

$$W\{ij\} = W[n_1, n_2, \ldots, n_{k-1}, n_k - 1, n_{k+1} - 1, \ldots, n_{i-1} - 1, n_{i+1} - 1, \ldots, n_n - 1] \tag{29}$$

Therefore, the permanent of $W\{ij\}$ can be written as

$$\begin{aligned}\mathrm{perm}(W\{ij\}) &= \left(\prod_{i'=1}^{k-1}(n_{i'}+1-i')\right)\left(\prod_{i'=k}^{i-1}(n_{i'}-1+1-i')\right)\left(\prod_{i'=i+1}^{n}(n_{i'}+1-1-(i'-1))\right) \\ &= \left(\prod_{i'=1}^{k-1}(n_{i'}+1-i')\right)\left(\prod_{i'=k}^{i-1}(n_{i'}-i')\right)\left(\prod_{i'=i+1}^{n}(n_{i'}+1-i')\right) \\ &= \frac{\mathrm{perm}(W)}{(n_i+1-i)}\prod_{i'=k}^{i-1}\frac{(n_{i'}-i')}{n_{i'}+1-i'} \end{aligned} \tag{30}$$

This gives for $P_{ij}$:

$$P_{ij} = \frac{1}{(n_i+1-i)}\prod_{i'=k}^{i-1}\frac{(n_{i'}-i')}{n_{i'}+1-i'} \tag{31}$$

We can compare this result with that of one row below (row $i+1$):

$$P_{(i+1)j} = \frac{1}{(n_{i+1}+1-(i+1))} \prod_{i'=k}^{i} \frac{(n_{i'}-i')}{n_{i'}+1-i'} = \frac{P_{ij}(n_i+1-i)}{(n_{i+1}-i)} \frac{(n_i-i)}{n_i+1-i} = P_{ij} \frac{n_i-i}{(n_{i+1}-i)} \tag{32}$$

Therefore, we have following recursive relations

$$P_{ij} = \begin{cases} 0, & \text{if } W_{ij}=0 \\ \frac{1}{n_i+1-i}, & \text{if } W_{ij}=1 \text{ and } [W_{(i-1)j}=0 \text{ or } i=1] \\ \left(\frac{n_{i-1}+1-i}{n_i+1-i}\right) P_{(i-1)j}, & \text{otherwise} \end{cases} \tag{33}$$

For the example given above, this relation gives the following $P$-matrix:

$$P = \begin{matrix} & e_1 & e_2 & e_3 & e_4 & e_5 \\ s'_1 = s_1 & \frac{1}{2} & \frac{1}{2} & 0 & 0 & 0 \\ s'_2 = s_3 & \frac{1}{4} & \frac{1}{4} & \frac{1}{2} & 0 & 0 \\ s_3 = s_2 & \frac{1}{8} & \frac{1}{8} & \frac{1}{4} & \frac{1}{2} & 0 \\ s'_4 = s_4 & \frac{1}{8} & \frac{1}{8} & \frac{1}{4} & \frac{1}{2} & 0 \\ s'_5 = s_5 & 0 & 0 & 0 & 0 & 1 \end{matrix}$$

This $\mathcal{O}(n^2)$ algorithm can be done within a second for $n \leq 3500$, bigger than any foreseeable RETIS simulation, without even leveraging the block-diagonalization. One could swap again the second and third row to get them ordered according to the original $s_i$-states, though there is in principle no need for this. This is because it is irrelevant to connect the existing states to the ensembles in which they were originally created.

# 5 Kramers' theory

For Langevin dynamics, Kramers' relation provides a way to improve upon transition state theory via an approximate expression for the transmission coefficient:

$$\kappa = (1/\omega_b)\left(-\gamma/2 + \sqrt{\gamma^2/4 + w_b^2}\right) \tag{34}$$

Here, $\gamma$ is the friction coefficient of the Langevin dynamics and $\omega_b = \sqrt{k/m}$ with $m$ the particle's mass and $k$ the curvature along the reaction coordinate at the transition state. The rate constant is then the product of the transmission coefficient times the transition state theory expression for the rate:

$$k = \kappa k^{\mathrm{TST}} \tag{35}$$

For a one-dimensional motion along a coordinate $z$, the transition state theory expression can be expressed as:[6]

$$k^{\mathrm{TST}} = \sqrt{\frac{k_B T}{2\pi m}} \frac{e^{-\beta V(0)}}{\int_{-\infty}^{0} e^{-\beta V(z)} \mathrm{d}z} \tag{36}$$

where $V(\cdot)$ is the underlying potential, $T$ the temperature, $k_B$ the Boltzmann constant, and $\beta = 1/k_B T$. The transition state is here assumed to be located at $z = 0$ and the system is in state $A$, the reactant state, if $z < 0$.

The Kramers' approximation for the rate constant $k$ follows from Eqs. 34-36. However, other properties like crossing probabilities and the permeability through a membrane can be derived from the transmission coefficient as well.

The crossing probability $P_A(\lambda_B|\lambda_A)$ from interface $\lambda_A$ to interface $\lambda_B$ follows from the

main TIS/RETIS rate equation:

$$k = f_A P_A(\lambda_B|\lambda_A) \tag{37}$$

where $f_A$ is the conditional flux through $\lambda_A$ given the system is in state $A$. Here, $\lambda_A$ and $\lambda_B$ correspond to the first, $\lambda_0$, and last interface, $\lambda_M$, respectively. The flux $f_A$ through $\lambda_A$ is similar to $k^{\mathrm{TST}}$, the flux through the transition state without recrossing correction, as it counts all positive crossings and is based on the same normalization (integration over state $A$):

$$f_A = \sqrt{\frac{k_B T}{2\pi m}} \frac{e^{-\beta V(\lambda_A)}}{\int_{-\infty}^{0} e^{-\beta V(z)} \mathrm{d}z} \tag{38}$$

From Eqs. 34-38 we end up with an equation for the crossing probability:

$$P_A(\lambda_B|\lambda_A) = \frac{\kappa e^{-\beta V(0)}}{e^{-\beta V(\lambda_A)}} \tag{39}$$

Hence, based on the underlying potential and Kramers' expression, Eq. 34, one can obtain an approximate value for the crossing probability. Likewise, for a membrane system we can derive a Kramers' expression for the permeability $P$ starting from Eq. 18 in Ref. 4:

$$P = \frac{k}{(\rho_{\mathrm{ref}})_A} = \frac{f_A P_A(\lambda_B|\lambda_A)}{(\rho_{\mathrm{ref}})_A} \tag{40}$$

where $\rho_{\mathrm{ref}}$ refers to the probability density for a permeant at a location away from the membrane, $z_{\mathrm{ref}}$, where $V(\cdot)$ is considered to be flat, and the subscript $(\cdot)_A$ indicates that it is normalized over the reactant state region $A$:

$$(\rho_{\mathrm{ref}})_A = \frac{e^{-\beta V(z_{\mathrm{ref}})}}{\int_{-\infty}^{0} e^{-\beta V(z)} \mathrm{d}z} \tag{41}$$

Note that the integral in the denominator of Eqs. 38 and 41 is usually diverging since the

underlying potential $V(\cdot)$ is generally flat away from the barrier in a membrane system. Fortunately, this integral term cancels in Eq. 40:

$$P = \sqrt{\frac{k_B T}{2\pi m}} \left( \frac{e^{-\beta V(\lambda_A)}}{e^{-\beta V(z_{\mathrm{ref}})}} \right) P_A(\lambda_B|\lambda_A) = \sqrt{\frac{k_B T}{2\pi m}} \left( \frac{\kappa e^{-\beta V(0)}}{e^{-\beta V(z_{\mathrm{ref}})}} \right) \tag{42}$$

where in the second equality we substituted $P_A(\lambda_B|\lambda_A)$ using Eq. 39. Hence, based on Eq. 34 and Eq. 42, we can obtain a value for the permeability based on Kramers' theory.

The aforementioned equations can be generalized for multidimensional systems by replacing the $V(z)$ terms with the Landau free energy $F(z)$. That is, for one additional degree of freedom $y$:

$$F(z) = -k_B T \ln \left( \int e^{-\beta V(y,z)} \mathrm{d}y \right) \tag{43}$$

In addition, if multiple reaction channels yield competing parallel saddle points in the potential energy surface, these need to summed up as we will do in the next section.

## 5.1 Kramers' relation for crossing probability of a two-channel system

The potential energy surface described in Ref. 4 is the following

$$\begin{aligned} V(y,z) &= e^{-cz^2} \left( V_1 + A + A \sin\left(\frac{2\pi y}{L_y}\right) + B + B \cos\left(\frac{4\pi y}{Ly}\right) \right) \text{ with} \\ & A = (V_2 - V_1)/2,\ B = V_{\max}/2 - V_1/4 - V_2/4, \\ & V_1 = 10,\ V_2 = 11,\ V_{\max} = 20,\ c = 1,\ L_y = 6 \end{aligned} \tag{44}$$

Note that the potential is periodic along the $y$-direction such that $V(y,z) = V(y+L_y,z)$ and that it is zero in the limit $|z| \to \infty$. Further, the following mass, Langevin friction coefficient and thermodynamic parameters were set in dimensionless reduced units: $\gamma = 5, \quad T = m =$

$k_B = \beta = 1$. The first and last interfaces were set at: $\lambda_A = -1.5$ and $\lambda_B = 1.2$. In this case, we have two saddle points at $(-L_y/4, 0)$ and at $(+L_y/4, 0)$ where the former is slightly lower in potential energy by $1k_BT$ ($V_1$ and $V_2$, respectively). The curvatures can be obtained by applying a second order Taylor expansion around $z = 0$:

$$V(-L_y/4, z) \approx V_1 - cV_1z^2 \Rightarrow k_1 = 2cV_1$$

$$V(+L_y/4, z) \approx V_2 - cV_2z^2 \Rightarrow k_2 = 2cV_2$$

which gives $w_{b,1} = \sqrt{20}$ and $w_{b,2} = \sqrt{22}$. As a result $\kappa_1 = 0.5866, \quad \kappa_2 = 0.6002$ via Eq. 34. From this we can compute the crossing probability based on essentially Eq. 39, but using the Landau free energy, $F(\cdot)$, by Eq. 43, instead of the potential energy, $V(\cdot)$, and using both transmission coefficients for the parts along the orthogonal coordinate, $y$, where they are relevant:

$$P_A(\lambda_B|\lambda_A) \approx \frac{\kappa_1 \int_{-3}^{0} e^{-\beta V(y,0)} \mathrm{d}y + \kappa_2 \int_{0}^{3} e^{-\beta V(y,0)} \mathrm{d}y}{\int_{-3}^{3} e^{-\beta V(y,\lambda_A)} \mathrm{d}y} = 1.61 \cdot 10^{-5} \tag{45}$$

where the integrals over $y$ are taken over one period. Note that the system in Ref. 4 actually contains 3 particles that move in this 2D potential energy surface such that the dimension of the system is actually 6. However, since we follow one single target permeant and the other particles are assumed to have no influence on the target (the interparticle interaction was set to 0[4]), the effective dimension for our analysis is 2 with coordinates $y$ and $z$.

The permeability then follows from Eq. 42 with $V(\cdot)$ replaced by $F(\cdot)$, where we used the expression based on the crossing probability to have the effect of the two different transmission coefficients directly included:

$$P = \sqrt{\frac{k_BT}{2\pi m}} \left( \frac{\int_{-3}^{3} e^{-\beta V(y,\lambda_A)} \mathrm{d}y}{\int_{-3}^{3} e^{-\beta V(y,z_{\mathrm{ref}})} \mathrm{d}y} \right) P_A(\lambda_B|\lambda_A) = \frac{1}{6} \sqrt{\frac{k_BT}{2\pi m}} \left( \int_{-3}^{3} e^{-\beta V(y,\lambda_A)} \mathrm{d}y \right) P_A(\lambda_B|\lambda_A) = 1.37 \cdot 10^{-6} \tag{46}$$

where we assumed that $z_{\text{ref}}$ is taken far away from the membrane at $z = 0$ such that $z_{\text{ref}} \ll 0$ and $V(y, z_{\text{ref}}) \approx 0$.

### 5.2 Kramers' relation for crossing probability of double well potential

The double well potential is given by[5]

$$V(z) = k_1 z^4 - k_2 z^2 \text{ with } k_1 = 1, \quad k_2 = 2 \tag{47}$$

which has a transition state at $z = 0$ and minima at $z = -1$ and $z = 1$. Further is given that $T = 0.07$ and $k_B = m = 1$ such that the transition state theory expression for the rate, Eq. 36, equals:[5] $k^{\text{TST}} = 2.776 \cdot 10^{-7}$.

The curvature at the transition state equals $2k_2 = 4$ such that $w_b = 2$. Together with the friction coefficient of $\gamma = 0.3$, Kramers' relation, Eq. 34, provides a transmission coefficient: $\kappa = 0.9278$. Henceforth, by Eq. 35 the rate constant based on Kramers' theory equals: $k = 2.58 \cdot 10^{-7}$.

The crossing probability follows from Eq. 39 where in this case $\lambda_A = -0.99$.[3] From the previously determined value for $\kappa$, we get: $P_A(\lambda_B|\lambda_A) = 5.83 \cdot 10^{-7}$

## 6 Computational efficiencies

In this paper, the computational efficiency is defined as

$$\text{efficiency} = \frac{1}{\tau^{\text{eff}}} \tag{48}$$

where $\tau^{\text{eff}}$ is the efficiency time,[7] which is equal to the computational cost that is needed to get a statistical relative error equal to 1 for the property that is computed. Here, $\tau^{\text{eff}}$ could be expressed as the number of MD steps in path sampling simulations of large systems or

path sampling simulations based on Ab Initio MD where the number of force calculations completely determines the total CPU cost. Expressing the efficiency time is this way has the advantage that it is hardware independent. In this article, however, we express the efficiency time in actual CPU- or wall-time seconds in order to include also the computational cost for calculating the permanents in the replica exchange move.

When a simulation is completed after a certain time $\tau$ and the relative error $\epsilon$ has been obtained via, e.g. independent runs, block averaging or bootstrapping, the efficiency time is estimated by

$$\tau^{\text{eff}} = \epsilon^2 \tau \tag{49}$$

Note that for serial simulations this property is in principle independent of the simulation length $\tau$. If we run the simulation longer by a certain factor, the error should reduce by the square root of this factor such that $\tau^{\text{eff}}$ remains unchanged. However, we should realize that there is a rather large statistical uncertainty in the estimated values for $\tau^{\text{eff}}$ due the fact that the statistical error in the error is generally large.

In the following, unless stated otherwise, we will refer to the CPU-time and CPU-based efficiency time when referring to $\tau$ and $\tau^{\text{eff}}$. However, let us shortly discuss the wall-time efficiency that follows from the same equation, Eq. 49, but with $\tau$ being the wall-time instead of CPU-time. In all our simulations, we fixed the wall-time to $5\times12$ hours with 5 independent runs. So the wall-time is constant and independent to the number of workers that is used. However, with $K$ workers instead of 1, the CPU-time increases by a factor $K$. This means that if the error would follow the same trend as in a serial run, the use of $K$ instead of 1 worker would result in a $\sqrt{K}$ reduction of the error. Yet, with $\tau$ in Eq. 49 being the wall-time instead of CPU-time, the reduction in the error is not canceled by an increase in $\tau$ and the

efficiency, Eq. 48, would increase linearly with $K$. This would mean that we can write:

$$\text{efficiency(wall-time)} = K \times \text{efficiency(CPU-time)} \tag{50}$$

if the parallel run uses the total CPU-time as effectively as a serial simulation that runs $K \times 5 \times 12$ hours long. However, our parallel algorithm will introduce changes in the relative CPU-time that is used for MC moves in the different ensembles. This effect was investigated for the memoryless single variable stochastic (MSVS) process. In the next subsection, we give the meaning and derivation of the continuous curves shown in Fig. 1 of the main article.

## 6.1 Theoretical efficiencies for the MSVS process

The efficiency time can also be calculated for for specific parts of the calculation. In specific, TIS/RETIS consists of different path ensemble simulations that compute a local crossing probability. In the path ensemble $[k^+]$ which consists of paths that at least cross $\lambda_k$, this local crossing probability equals the fraction of paths that cross $\lambda_{k+1}$ as well. Based on the expected error in the local crossing probability, the CPU-based efficiency time of ensemble $[k^+]$ can be expressed as:[7]

$$\tau_k^{\text{eff}} = \frac{1 - p_k}{p_k} \mathcal{N}_k \xi_k L_k \tag{51}$$

where $p_k$ is the local crossing probability of ensemble $[k^+]$, $L_k$ is the average path length (expressed in MD steps or CPU seconds), and $\xi_k$ is the ratio of the average cost of a MC move to $L_k$. In other words, $\xi_k L_k$ is the average computational cost for doing a MC move (creation of a trial path that might then be accepted or rejected). Finally, $\mathcal{N}_k$ is a measure of the effective correlations between MC moves also called the "statistical inefficiency". Paths can be correlated due to rejections, which implies that the old path is recounted, or because of similarities between accepted paths. In practice, $\mathcal{N}_k$ tends to be significantly larger than 1 while $\xi_k$ is often smaller than 1 as many rejections occur without that a trial path needs

to be fully completed. In addition, some MC moves like the replica exchange move or the time-reversal move do not require any MD steps.

In the following, we will neglect the effect that the replica exchange moves have on the errors and on the CPU-time. Under this assumption, the successive MC moves are completely independent. In addition, the ensemble moves are memoryless (hence $\mathcal{N} = 1$). The overall error can thus be computed from the errors in the individual ensembles using standard error propagation rules for independent estimates. Except for the replica exchange part, the MSVS simulation is rejection-free such that we also have $\xi = 1$. In addition, the random artificial MD time for a path in ensemble in ensemble $[k^+]$ was on average $0.1\,k + 0.1$ seconds. To simplify our analysis, we neglect the final 0.1 addition, and state that $L_k = ak$ with $a = 0.1$. Finally, we fixed the local crossing probability to $p_k = p = 1/10$ for all ensembles $[k^+]$ such that

$$\tau_k^{\rm eff} = a\frac{1-p}{p}k \tag{52}$$

The relative error in estimate of the local crossing probability of ensemble $[k^+]$ follows from Eq. 49:

$$\epsilon_k = \sqrt{\frac{\tau_k^{\rm eff}}{\tau_k}} \tag{53}$$

with $\tau_k$ the CPU-time that is spend to ensemble $[k^+]$. Given a certain division of the total simulation time $\tau$ into the times $(\tau_0, \tau_1, \ldots, \tau_{N-1})$, we can compute the total efficiency time by Eq. 49 with

$$\epsilon^2 = \sum_{k=0}^{N-1} \epsilon_k^2 = \sum_{k=0}^{N-1} \frac{\tau_k^{\rm eff}}{\tau_k} \quad \text{and} \quad \tau = \sum_{k=0}^{N-1} \tau_k \tag{54}$$

The first expression is the standard error propagation rule for the error in a final estimate that is obtained from a product of independent estimates.

Now let us first consider standard TIS or the $N = K$ case. In this simulation we would have an equal number of workers as ensembles. Each worker is solely designated to a single ensemble such that an equal amount of CPU-time is spend per ensemble when the simulation is stopped. So we can simply put $\tau_k = 1$ such that $\tau = N$ and

$$\epsilon^2 = \sum_{k=0}^{N-1} \tau_k^{\text{eff}} = a\frac{1-p}{p}\sum_{k=0}^{N-1} k = a\frac{1-p}{p}\frac{1}{2}(N-1)N \approx \frac{a}{2}\frac{1-p}{p}N^2 \tag{55}$$

where in the last equality we assumed $N \gg 1$. The efficiency time for TIS is hence

$$\tau^{\text{eff}} \approx \frac{1}{2}a\frac{1-p}{p}N^3, \qquad \text{for TIS or } K = N \tag{56}$$

For serial RETIS, each ensemble is updated by a MC move before a next cycle of moves is started. As a result, in each ensemble the same number of MC moves are carried out such that $\tau_k \propto L_k \propto k$. By taking $\tau_k = k$, we get that $\tau = (N-1)N/2 \approx N^2/2$ and

$$\epsilon^2 = \sum_{k=0}^{N-1} \frac{\tau_k^{\text{eff}}}{\tau_k} = aN\frac{1-p}{p} \tag{57}$$

and the CPU-based efficiency time is exactly the same

$$\tau^{\text{eff}} \approx \frac{1}{2}a\frac{1-p}{p}N^3, \qquad \text{for RETIS or } K = 1 \tag{58}$$

This is in agreement with Ref. 7 which stated that an equal division of CPU-time or aiming for the same error in each ensemble gives the same efficiency. Since the local crossing probability is the same for each ensemble, $p_k = p$, aiming for the same error in each ensemble is equivalent to having the same number of MC moves per ensemble (if the statistical inefficiencies, $\mathcal{N}_k$, are the same). The optimal division of CPU-time over the different ensembles

is, however, $\tau_k \propto \sqrt{\tau_k^{\text{eff}}}$.[7] By taking $\tau_k = \sqrt{k}$, the total CPU-time becomes

$$\tau = \sum_{k=0}^{N-1} \sqrt{k} \approx \int_0^N \sqrt{x}\mathrm{d}x = \frac{2}{3}N^{3/2} \tag{59}$$

and the total error

$$\epsilon^2 = \sum_{k=0}^{N-1} \frac{\tau_k^{\text{eff}}}{\tau_k} = a\frac{1-p}{p}\sum_{k=0}^{N-1}\sqrt{k} \approx a\frac{1-p}{p}\frac{2}{3}N^{3/2} \tag{60}$$

which by Eq. 49 results in a slightly lower efficiency time than for TIS/RETIS:

$$\tau^{\text{eff}} \approx \frac{4}{9}a\frac{1-p}{p}N^3, \qquad \text{for an optimal division} \tag{61}$$

Based on $a = p = 0.1$ and $N = 50$, the efficiency times are $\tau^{\text{eff}} = 56250$ for TIS/RETIS and $\tau^{\text{eff}} = 50000$ for the optimal division. Naturally, the corresponding CPU-time efficiencies by Eq. 48 are 1/56250 and 1/50000. Furthermore, based on Eq. 50, the optimal wall-time efficiency and the optimal TIS/RETIS wall-time efficiency are given by $K/50000$ and $K/56250$, respectively. These are the continuous black and purple curves in Fig.1d of the main article.

It is interesting to observe that the optimal TIS/RETIS CPU-time efficiency is only 12.5% lower than the optimal CPU-time efficiency. This seems to suggest that it is difficult to improve the CPU-time efficiency of TIS and RETIS unless the division of CPU-time is exactly targeted to do so. On the other hand, one can easily get a much worse CPU-time efficiency when errors in some ensembles are reduced to unnecessary small values while the other ensemble errors are ignored. Based on the fact that $\tau_k \propto \sqrt{\tau_k^{\text{eff}}}$ gives the optimum, the optimum division of MC moves is obtained when in ensembles $[k^+]$ the number of MC moves is proportional to $\sqrt{\tau_k^{\text{eff}}}/L_k$. For the MSVS system this means that the number of executed MC moves in each ensemble should optimally be taken as $\propto 1/\sqrt{k}$ for $k = 1, 2, \ldots, M-1$ (to account for $k = 0$ we should have kept the neglected 0.1 addition in the path length

to avoid divergence). This means that it is actually good to execute more MC moves at the lower rank ensembles (low $k$) than at the higher rank (high $k$). However, this should not be exaggerated since too many MC moves in the low ranked ensembles will just result in inefficient use of CPU-time as discussed above. Based on the numerical sampling ratios, we determined the CPU-time spend in each ensemble, $\tau_k$, by multiplying these ratios by $L_k = ak$. We then estimated the error based on Eqs. 54 and 52. The resulting efficiency, based on the actual sampling ratios of ∞RETIS, turned out to give a slightly better CPU-time efficiency than that of TIS/RETIS for $15 \leq K \leq 45$, shown by the purple dashed line in Fig. 1d. The resulting wall-time efficiencies of this hybrid theoretical/numerical result is shown by the purple dots if Fig.1d as well. This shows that ∞RETIS can actually improve both the CPU- and wall-time efficiency compared to TIS/RETIS. The latter is expected based on the brute force principle that more CPU power is used per second. The former is more subtle and related to the fact that ∞RETIS leads to a more efficient distribution of the CPU-time among the different ensembles compared to TIS or RETIS.

# 7 Additional simulation results

## 7.1 Ratios of channels crossings

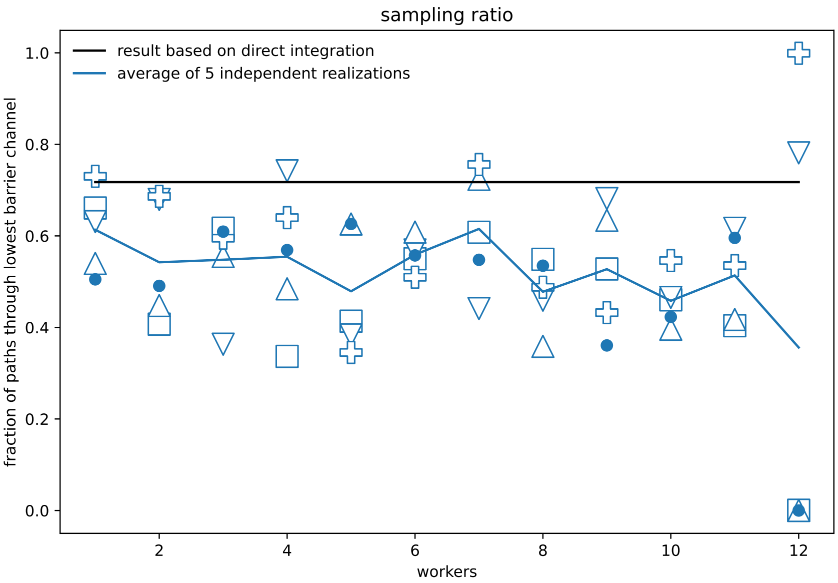


Figure 1: The ratio of first crossings points for the last ensemble in the more favorable channel. The blue icons shows the sampled ratio for each simulation, the blue line is the average of 5 simulations for each amount of workers and the black line is the expected value from direct integration of $\exp(-\beta V(y,z))$ over $y$ with $z$ fixed at $z = \lambda_{10} = -0.2$. This gives an approximate theoretical value of 0.71. The exact value is probably slightly less as the integrated probability density does not correct for phase points that should not be counted as they are actually lying on a path coming from state $B$ rather than $A$. Still, a majority of simulations provide a fraction that is slightly too low, which is likely an effect of the initial conditions. As reported in Ref. 4, this ratio requires many MC moves to converge without the added MC moves introduced in that paper (mirror-move and target-swap move). These added moves are in principle perfectly compatible with the new replica exchange method, but those were not implemented yet in this work. Therefore, we see the same slow convergence for all of our simulations. Still, the $K < N$ simulations are strikingly better than the $K = N = 12$ case where no replica exchange moves were performed. For 12 workers, the 3 icons overlapping at 0.0 and another simulation showing a fraction equal to 1.0 are the result of the known ergodicity issues of the TIS algorithm due to the lack of swapping moves.

# 8 Code availability

All simulation code for this paper is publicly available at doi.org/10.5281/zenodo.6977013. Fair warning; this code is not user-friendly and highly optimized for our specific hardware and output requirements. Instead we would advice everyone to instead use the examples of the infretis github (https://github.com/infretis/infretis).

This code was purely developed to verify the soundness and performance of the algorithm. It uses python multiprocessing with a custom communication code, mimicking MPI, to let the workers run arbitrary python code. It is limited to running on a single machine as no network interface was written for the communication layer and it assumes all files to be accessible by all processes. It uses internal calls to PyRETIS[8] to run the MD for the two-channel and 1D-wirefencing simulations and no current support is present for dealing with external MD engines as with more mature path-sampling codes, like OPS[9] and PyRETIS.[8] It also does not write any data, which means the simulation can not be reanalyzed after it has completed.

A new project has started at https://github.com/infretis/infretis, which will rewrite this custom code to a more user-friendly software. It will add the file handling, use Dask[10] to manage the parallelization which allows for out-of-machine scaling, and will include external MD packages as is common in path sampling codes. Initial examples of the Dask integration can be found in the example directory of that git repository.

## References


(1) Dellago, C.; Bolhuis, P. G.; Chandler, D. Efficient transition path sampling: Application to Lennard-Jones cluster rearrangements. *J. Chem. Phys.* **1998**, *108*, 9236–9245.

(2) Riccardi, E.; Dahlen, O.; van Erp, T. S. Fast decorrelating Monte Carlo moves for efficient path sampling. *J. Phys. Chem. Lett.* **2017**, *8*, 4456–4460.

(3) Zhang, D. T.; Riccardi, E.; van Erp, T. S. Enhanced path sampling using subtrajectory Monte Carlo moves. Preprint. 2022; `https://doi.org/10.48550/arXiv.2210.07026`.

(4) Ghysels, A.; Roet, S.; Davoudi, S.; van Erp, T. S. Exact non-Markovian permeability from rare event simulations. *Phys. Rev. Research* **2021**, *3*, 033068.

(5) van Erp, T. S. Dynamical rare event simulation techniques for equilibrium and nonequilibrium systems. *Adv. Chem. Phys.* **2012**, *151*, 27.

(6) Frenkel, D.; Smit, B. *Understanding molecular simulations from algorithms to applications*; Academic press: San Diego, California, U.S.A., 2002.

(7) van Erp, T. S. Efficiency analysis of reaction rate calculation methods using analytical models I: The two-dimensional sharp barrier. *J. Chem. Phys.* **2006**, *125*, 174106.

(8) Riccardi, E.; Lervik, A.; Roet, S.; Aarøen, O.; van Erp, T. S. PyRETIS 2: An improbability drive for rare events. *J. Comput. Chem.* **2020**, *41*, 370–377.

(9) Swenson, D. W. H.; Prinz, J.-H.; Noe, F.; Chodera, J. D.; Bolhuis, P. G. OpenPathSampling: A Python Framework for Path Sampling Simulations. 2. Building and Customizing Path Ensembles and Sample Schemes. *J. Chem. Theory Comput.* **2019**, *15*, 837–856.

(10) Dask Development Team, Dask: Library for dynamic task scheduling. 2016.
